# *Bacillus subtilis* YlxR, Which Is Involved in Glucose-Responsive Metabolic Changes, Regulates Expression of *tsaD* for Protein Quality Control of Pyruvate Dehydrogenase

**DOI:** 10.3389/fmicb.2019.00923

**Published:** 2019-05-01

**Authors:** Mitsuo Ogura, Tsutomu Sato, Kimihiro Abe

**Affiliations:** ^1^Institute of Oceanic Research and Development, Tokai University, Shizuoka, Japan; ^2^Department of Frontier Bioscience, Hosei University, Koganei, Japan; ^3^Research Center for Micro-Nano Technology, Hosei University, Koganei, Japan

**Keywords:** protein lysine acetylation, transposon mutagenesis, translational control, RNA polymerase, universal tRNA modification

## Abstract

Glucose is the most favorable carbon source for many bacteria, which have several glucose-responsive gene networks. Recently, we found that in *Bacillus subtilis* glucose induces the expression of the extracellular sigma factor genes *sigX* and *sigM* through the acetylation of CshA (RNA helicase), which associates with RNA polymerase (RNAP). We performed a transposon mutagenesis screen for mutants with no glucose induction (GI) of *sigX-lacZ*. While screening for such mutants, we recently found that the GI of *sigX/M* involves YlxR, a nucleoid-associated protein (NAP) that regulates nearly 400 genes, including metabolic genes. It has been shown that acetylated CshA positively regulates expression of *ylxR*-containing operon. Here, we report additional mutations in *yqfO* or *tsaD* required for the GI of *sigX*. YqfO contains a universally conserved domain with unknown function. YqfO and YlxR were found to regulate expression of the *tsaEBD*-containing operon. Mutational analysis using *lacZ* fusions revealed the adenine-rich *cis-*element for YlxR. TsaD is a component of the TsaEBD enzyme required for the synthesis of threonylcarbamoyl adenosine (t^6^A). The t^6^A modification of tRNA is universal across the three domains of life. Western blot analysis showed that the *tsaD* mutation in the presence of glucose reduced levels of soluble PdhA, PdhB, and PdhD, which are subunits of the pyruvate dehydrogenase complex (PDHc). This resulted in severely defective PDHc function and thus reduced concentrations of cellular acetyl-CoA, a reaction product of PDHc and plausible source for CshA acetylation. Thus, we discuss a suggested glucose-responsive system (GRS) involving self-reinforcing CshA acetylation. This self-reinforcing pathway may contribute to the maintenance of the acetyl-CoA pool for protein acetylation.

## Introduction

Glucose is the most favorable carbon source for many bacteria, and these bacteria have several glucose-responsive networks (Deutscher, 2008). In Gram-positive bacteria, including *Bacillus subtilis*, the transcription factor CcpA is the master regulator for the carbon catabolite regulation (Deutscher, 2008; Fujita, 2009). The incorporation of glucose in the bacterial culture medium results in an increase of the metabolite fructose 1,6-bisphosphate, which triggers the phosphorylation of Ser46 of HPr, a phosphocarrier protein in the sugar phosphotransferase system (P-Ser-HPr). P-Ser-HPr associates with and activates CcpA, leading to global positive and negative effects on the transcriptional network. Moreover, there are several additional glucose-responsive transcription factors, such as CcpC, CcpN, CggR, and GlcT (Fujita, 2009). In *Escherichia coli* catabolite gene-activator protein CAP has been conventionally considered a transcription factor responding to glucose. However, recent genomic analyses led to an idea, that CAP is also a nucleoid-associated protein (NAP, Dillon and Dorman, 2010; Sandhya et al., 2015).

Accumulated studies identified proteins called as NAP which are not structurally related to histones but have similar functions to histones in bacteria (Drlica and Rouviere-Yaniv, 1987; Browning et al., 2010; Dillon and Dorman, 2010). NAPs have many roles in transcription, recombination including phage-infection, and chromosome condensation, rearrangement, maintenance, and segregation (Dillon and Dorman, 2010). NAPs generally have non-specific DNA-binding activity or recognize local DNA structure (Browning et al., 2010). However, NAPs, such as Fis and IHF bind to specific DNA sequences (Azam and Ishihama, 1999). The modes of transcriptional regulation of NAPs are diverse, for example, H-NS inhibits RNA polymerase (RNAP) progression on DNA, while Fis regulates transcription through various modes of interaction with RNAP (Dillon and Dorman, 2010). YlxR is a NAP of *B. subtilis*, which either positively or negatively regulates approximately 400 genes (Ogura and Kanesaki, 2018). Furthermore, YlxR is shown to be involved in the glucose induction (GI) of various genes, including *sigX* and *sigM*, which encode extracellular function (ECF) sigma factors SigX and SigM, respectively (Helmann, 2016; Ogura and Asai, 2016; Ogura and Kanesaki, 2018).

**FIGURE 1:**
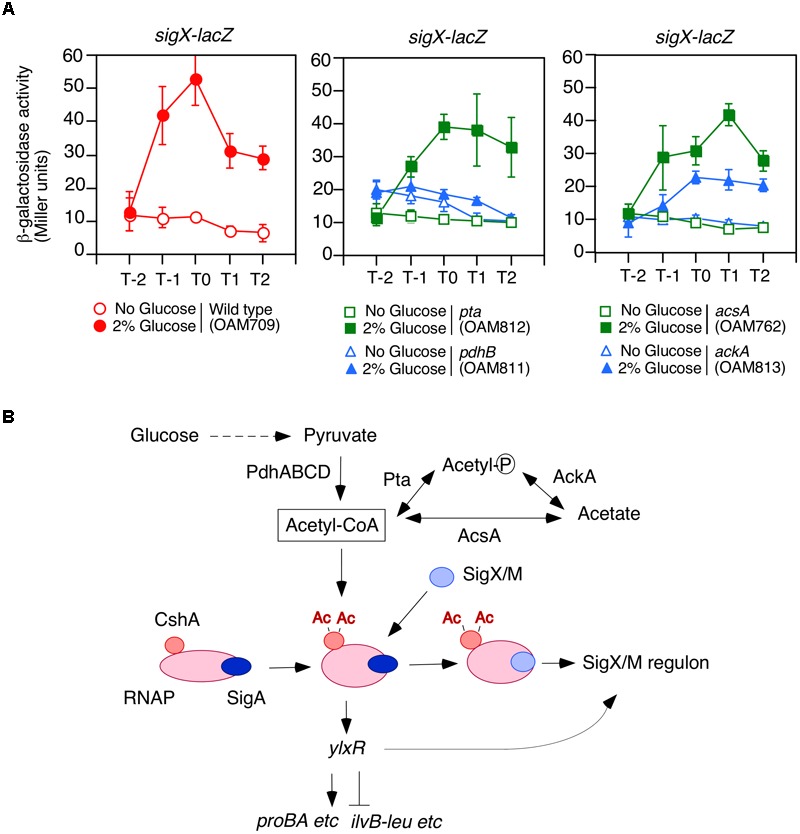
Requirement of acetyl-CoA produced by PDHc for GI of *sigX-lacZ*. **(A)** Expression of *sigX-lacZ* in the mutants. Means of the β-Gal activities from three independent experiments and the standard deviations are shown. The *x* axis represents the growth time in hours relative to the end of vegetative growth (T0). All strains are the derivatives of wild-type OAM709 and the relevant genotype is indicated below the panel. **(B)** Model of GI of *sigX*/*sigM* and acetyl-CoA metabolism. Glucose addition stimulates the acetylation of CshA (Ogura and Asai, 2016). CshA has been shown to associate with RNAP. RNAP with acetylated CshA may stimulate the replacement of σ^X/M^ for σ^A^ in the RNAP holoenzyme. σ^A^ -associated RNAP holoenzyme with acetylated CshA stimulate the transcription of *ylxR* (Ogura and Kanesaki, 2018). YlxR regulates many genes and changes the cellular metabolic state, which may lead to induction of the SigX/M regulons. Ac, acetyl moiety; PdhABCD, pyruvate dehydrogenase (PDH); Pta, phosphotransacetylase; AcsA, acetyl-CoA syntheatse; AckA, acetate kinase. “P” in the circle indicates phosphate residue.

Several hundreds of lysine-acetylated proteins have been identified in many bacteria (Ouidir et al., 2016; Carabetta and Cristea, 2017). Glucose addition to the medium often induced protein acetylation in bacteria, such as *E. coli* and *B. subtilis* (Lima et al., 2011; Kosono et al., 2015; Schilling et al., 2015). Proteomic analysis of *B. subtilis* revealed that CshA, one of the DEAD-box helicases, is acetylated (Lehnik-Habrink et al., 2013; Kosono et al., 2015). We recently found that addition of glucose stimulated lysine acetylation of CshA (Ogura and Asai, 2016). CshA is also known to associate with RNAP (Delumeau et al., 2011). The association of acetylated CshA with RNAP would enhance its affinity to SigX and SigM (Figure 1; Helmann, 2016; Ogura and Asai, 2016). This leads to GI of *sigX* and *sigM* (Shiwa et al., 2015; Ogura and Asai, 2016). In most cases, ECF sigma factors are subject to membrane-embedded anti-sigma factors, which trap a cognate ECF sigma factor, leading to inactivation of the ECF sigma factor (Helmann, 2016). However, CshA-dependent GI of SigX/M is not under control of anti-sigma factors (Ogura and Asai, 2016). The GI of *sigX* caused by acetylation of CshA was susceptible to disruption by the mutation of genes encoding pyruvate dehydrogenase (PDH), namely *pdhABCD* (Gao et al., 2002; Ogura and Asai, 2016). PDH consists of the multi-enzyme subunit (PDHc) and is an enormously large protein complex. The disruption of the *pdh* genes would result in the reduction of the intracellular acetyl-CoA pool, which is affected by the activity of PDH, that is, the conversion of pyruvate to acetyl-CoA. PDH has three enzymatic activities and three components: PDH [E1 (PdhA and PdhB)], dihydrolipoamide acetyltransferase [E2 (PdhC)], and lipoamide dehydrogenase [E3 (PdhD)] (Hodgson et al., 1983). Additionally, two genes are involved in acetyl-CoA metabolism through synthesis of acetyl-phosphate, *pta* encoding phosphotranacetylase and *ackA* encoding acetyl kinase. Moreover, with glucose as the carbon source cellular concentrations of acetyl-phosphate decrease in the *pta* strain, while it accumulates in the *ackA* strain (Klein et al., 2007) and both mutations have severe effects on the *B. subtilis* acetylated proteome (Kosono et al., 2015).

The transposon mutagenesis screen for mutants with no GI of *sigX-lacZ* revealed *yqfO* and *tsaD* in addition to the previously analyzed *cshA* and *ylxR* genes (Ogura and Asai, 2016; Ogura and Kanesaki, 2018). Here we analyzed *yqfO* and *tsaD* in detail. TsaD is a component of a tRNA modification enzyme that is required for the synthesis of threonylcarbamoyl adenosine (t^6^A) (Thiaville et al., 2014, 2015). The CshA acetylation induced *ylxR*-containing operon expression (Ogura and Kanesaki, 2018) and YlxR and YqfO regulated the *tsaD*-containing operon. In the *tsaD* mutant, the soluble PDHc subunits were markedly reduced in the presence of glucose. This would contribute to the observed low intracellular acetyl-CoA pool and result in reduced CshA acetylation. The finding that the disruption of *tsaD* decreases the soluble PDHc subunits suggests a relationship between the lack of t^6^A and protein quality control.

## Materials and Methods

### Strains, Media, and β-Galactosidase Analysis

All *B. subtilis* strains used in this study are listed in Table 1 and Supplementary Table S1. One-step competence medium (MC; Kunst et al., 1994), Schaeffer’s sporulation medium (Schaeffer et al., 1965), and Luria-Bertani (LB) medium (Difco, Lennox) were used. Antibiotic concentrations were described previously (Ogura and Tanaka, 1996; Ogura et al., 1997). Synthetic oligonucleotides were commercially prepared by Tsukuba Oligo Service (Ibaraki, Japan) and are listed in Supplementary Table S2. Methods of β-galactosidase analysis using ONPG(2-Nitrophenyl-β-D-galactopyranoside) were previously described (Ogura and Tanaka, 1996). β-galactosidase analysis using CRPG (Chlorophenol red β-D-galactopyranoside, Roche, Germany) was performed using method similar to those used for ONPG. Since CRPG is the red pigment, 0.7 ml of Z-buffer with 0.2 ml of CRPG solution (4 mg/ml in Z-buffer) and 0.5 ml of 1 M Na_2_CO_3_ in water was mixed and used for measurement of OD_570_ as a blank control. To calculate Miller units, OD_550_ values were measured using cell suspension samples processed in parallel.

**Table 1:** Strains and plasmids used in the study.

Strain/Plasmid	Genotype	References or source
168	*trpC2*	Laboratory stock
OAM734	*trpC2 tsaD*::Tn (Km^r^)	This study
OAM735	*trpC2 ylxR* (Em^r^ *lacZ::Tc^r^)*	Ogura and Kanesaki, 2018
YqfOd	*trpC2 yqfO* (Em^r^ *lacZ)*	BSORF
OAM737	*trpC2 yqfO* (Em^r^ *lacZ::Tc^r^)*	This study
501-76	*trpC2 pdhB* (Km^r^)	Gao et al., 2002
OAM709	*trpC2 thrC::sigX-lacZ* (-43 to +262^1^, Em^r^)	Ogura and Asai, 2016
OAM811	*trpC2 thrC::sigX-lacZ* (-43 to +262, Em^r^) *pdhB* (Km^r^)	This study
OAM812	*trpC2 thrC::sigX-lacZ* (-43 to +262, Em^r^) *pta* (Tc^r^)	This study
OAM762	*trpC2 thrC::sigX-lacZ* (-43 to +262, Em^r^) *acsA* (Tc^r^)	This study
OAM813	*trpC2 thrC::sigX-lacZ* (-43 to +262, Em^r^) *ackA* (Sp^r^)	This study
OAM767	*trpC2 thrC::sigX-lacZ* (-43 to +262, Em^r^) *yqfO* (Em^r^*lacZ*::Tc^r^)	This study
OAM874	*trpC2 thrC::sigX-lacZ* (-43 to +262, Em^r^) *tsaD*::Tn (Km^r^)	This study
OAM764	*trpC2 thrC::sigX-lacZ* (-43 to +262, Em^r^) *tsaB* (Tc^r^)	This study
OAM738	*trpC2 thrC*::*sigX-lacZ* (-43 to +262, Em^r^) *amyE*::Px*-yqfO* (Cm^r^) *yqfO* (Em^r^*lacZ*::Tc^r^)	This study
OAM739	*trpC2 thrC::sigX-lacZ* (-43 to +262, Em^r^) *amyE*::Px-*tsaD* (Cm^r^) *tsaD*::Tn (Km^r^)	This study
OAM850	*trpC2 amyE*::P*trmK-lacZ* (-295 to -1^2^, Cm^r^)	This study
OAM851	*trpC2 amyE*::P*trmK-lacZ* (-295 to -1, Cm^r^) *ylxR* (Km^r^)	This study
OAM747	*trpC2 thrC*::P*thiL-lacZ* (-500 to -1^2^, Sp^r^)	This study
OAM748	*trpC2 thrC*::P*thiL-lacZ* (-500 to -1, Sp^r^) *ylxR* (Em^r^ *lacZ*::Tc^r^)	This study
OAM749	*trpC2 thrC*::P*thiL-lacZ* (Sp^r^) *yqfO* (Em’ *lacZ*::*Tcr*)	This study
FU1019	*trpC2 lys amyE*::P*pdhA-lacZ* (-167/-47^1^, Cm^r^)	Tojo et al., 2010
OAM759	*trpC2 amyE*::P*pdhA-lacZ* (-161/-47, Cm^r^)	This study
OAM760	*trpC2 amyE*::*PpdhA-lacZ* (-167/-47, Cm^r^) *tsaD*::Tn (Km^r^)	This study
OAM761	*trpC2 pdhA*::pMUT-His-*pdhA* (Em^r^)	This study
OAM762	*trpC2 pdhA*::pMUT-His-*pdhA* (Em^r^) *tsaD*::Tn (Km^r^)	This study
OAM777	*trpC2 pdhB*::pMUT-His-*pdhB* (Em^r^)	This study
OAM778	*trpC2 pdhB*::pMUT-His-*pdhB* (Em^r^) *tsaD*::Tn (Km^r^)	This study
OAM779	*trpC2 pdhD*::pMUT-His-*pdhD* (Em^r^)	This study
OAM780	*trpC2 pdhD*::pMUT-His-*pdhD* (Em^r^) *tsaD*::Tn (Km^r^)	This study
Plasmid	Description	This study
pX	Amp^r^ *amyE::xylR* -Pxyl Cm^r^	Hori et al., 2002
pX-yqfO	Amp^r^ *amyE*::*xylR*-Pxyl-*yqfO* (*yqfO* ORF with its SD), Cm^r^	This study
pX-tsaD	Amp^r^ *amyE*::*xylR*-Pxyl-*tsaD (tsaD* ORF with its SD), Cm^r^	This study
pIS248	Amp^r^ *amyE::lacZ* Cm^r^	Tsukahara and Ogura, 2008
pIS-trmK	Amp^r^ *amyE*::P*trmK*-*lacZ* (-295 to -1^2^) Cm^r^	This study
pDG1729	Amp^r^ Em’ *thrC::lacZ* Sp^r^	Guérout-Fleury et al., 1996
pDG1729-thiL-Wt	Amp^r^ Em^r^ *thrC*::P*thiL*-*lacZ* (-500 to -1^2^) Sp^r^	This study
pLacZ::Tc	Amp^r^ *lacZ*::Tc^r^	Ogura et al., 2003
pTYB11-ylxR	Amp^r^ *intein-ylxR*	Ogura and Kanesaki, 2018
pMUTIN-His	Amp^r^ Em^r^ His-tag Pspac	Murayama et al., 2015
pMUT-His-pdhA	Amp^r^ Em^r^, C-terminal region of *pdhA*, His-tag Pspac	This study
pMUT-His-pdhB	Amp^r^ Em^r^, C-terminal region of *pdhB*, His-tag Pspac	This study
pMUT-His-pdhD	Amp^r^ Em^r^, C-terminal region of *pdhD*, His-tag Pspac	This study

*^*1*^Numbers indicate the nucleotide positions ralative to the transcription start point for *sigX-lacZ* and P*pdhA-lacZ*. ^*2*^Numbers indicate the nucleotide positions ralative to the translation start point for P*trmK-lacZ and* P*thiL-lacZ*. The rests of the strains and plasmids for deleted and mutated P*trmK-lacZ* and P*thiL-lacZ* fusions are listed in Supplementary Table S1.*

### Growth Condition

Strains were grown on a LB agar plate containing appropriate antibiotics at 37°C overnight. The cells were scraped and suspended in the sporulation medium. The suspension was inoculated into 50 ml sporulation medium (with or without 2% glucose) without antibiotics in a 200 ml flask. Klett value was adjusted around 10 units. The flask was gently shaken (110 reciprocation/min) at 37°C. Cell growth was monitored with Klett calorimeter (Klett Mfg. Co., Inc., New York, NY).

### Strain Construction

The *pta*::Tc^r^, *acsA*::Tc^r^, *tsaB*::Tc^r^, and *ackA*::Sp^r^ units were constructed using PCR. Briefly the upstream and downstream regions of the concerned genes and Tc^r^ from pBEST304 (Itaya, 1992) and Sp^r^ from pDG1729 (Guérout-Fleury et al., 1996) were amplified using the indicated primers (Supplementary Table S2) and then combined by PCR. These units were directly used for transformation of *B. subtilis* 168. From the resultant Tc^r^ and Sp^r^ strains total DNAs were taken. Those were used in PCR to confirm the expected chromosomal structure as template.

### Plasmid Construction

The plasmids used in this study are listed in Table 1 and Supplementary Table S1. pX-yqfO and pX-tsaD were constructed by cloning of the PCR products amplified with the oligonucleotide pairs, pX-yqfO-Spe/pX-yqfO-Bgl(*Spe*I/*Bgl*I digestion) and pX-gcp-Spe/pX-gcp-Bam (*Spe*I/*BamH*I digestion), respectively, into pX treated with *Spe*I/*BamH*I (Hori et al., 2002). To construct pDG1729-thiL, the PCR product amplified with the oligonucleotide pair pDG1729-gcp-E/pDG1729-gcp-B was digested with *EcoR*I/*BamH*I and cloned into pDG1729 treated with the same enzymes (Guérout-Fleury et al., 1996). To construct pIS-trmK, the PCR product amplified with the oligonucleotide pair yqfO-Eco/pIS-trmK-B was digested with *EcoR*I/*BamH*I and cloned into pIS284 treated with the same enzymes (Tsukahara and Ogura, 2008). To construct pMUT-His-pdhA, pMUT-His-pdhB, and pMUT-His-pdhD, the PCR products amplified with the oligonucleotide pairs, pdhA-F-E/pdhA-R-Xh, pdhB-F-E/pdhB-R-Xh, and pdhD-F-E/pdhD-R-Xh were digested with *EcoR*I/*Xho*I and cloned into pMUTIN-His treated with the same enzymes (Murayama et al., 2015).

### Western Blot Analysis

Cells were grown in 50 ml of sporulation medium with or without 2% glucose. At T1, cells were harvested and washed with 1 ml of TBS buffer (10 mM Tris–HCl pH 7.5, 150 mM NaCl) containing 1 mM PMSF. Cells were disrupted with French Pressure Cell and centrifuged (15000 × *g*) to obtain a cleared lysate. Western blot analysis was performed by a method similar to that described previously (Hata et al., 2001). Monoclonal mouse anti-His tag antibody was purchased from Medical and Biological Laboratories (Nagoya, Japan). Polyclonal rabbit anti-SigA antibody was previously described (Ogura, 2016). These antibodies were diluted (1/1000) in Can Get Signal solution 1 (ToYoBo, Tokyo, Japan), and Can Get Signal solution 2 (ToYoBo) was used for secondary antibody.

### Fractionation of Membrane and Aggregated Proteins

During preparation of cleared cell lysate, the cell pellets were obtained after centrifugation. The pellets were solubilized in 0.3 ml of 1% Triton X-100 in TBS buffer at 4°C for 30 min and then centrifuged (10000 × *g*) for 5 min. The obtained pellets were again solubilized in 0.3 ml of 0.5% Triton X-100 in TBS buffer at 4°C for 30 min and then centrifuged (10000 × *g*) for 5 min. Both solubilized fractions were mixed (membrane protein fractions). The obtained pellets were fractions of aggregated proteins and solubilized in 0.6 ml of 7 M urea, 100 mM DTT and 4% CHAPS at 4°C for 10 min. These fractions were examined by SDS-PAGE and western bolt analysis. The methods were similar to those described in a previous paper (Runde et al., 2014). In the study, mass spectrometric analysis showed only 1–4% contaminated membrane proteins in the aggregates fraction.

### Examination of the Concentration of Intracellular Acetyl-CoA

Cells were grown in 50 ml of sporulation medium with or without 2% glucose. At T0, 30 ml of cells was harvested and washed with TEN buffer (20 mM Tris–HCl pH 7.5, 1 mM EDTA, 150 mM NaCl, and 1 mM PMSF). Cells were disrupted with French Pressure Cell and centrifuged (15000 × *g*). The resulting cleared cell lysate was treated with a Deproteinizing Sample Preparation kit (Biovision, CA, United States). Measurement of the acetyl CoA concentration was performed with a PicoProbe Acetyl CoA Fluorometric Assay kit (Biovision).

## Results

### Requirement of Acetyl-CoA Produced by PDHc for Glucose Induction of *sigX-lacZ*

We observed that a mutation that disrupts *pdhC* abolishes the GI of *sigX-lacZ* (Ogura and Asai, 2016). A similar result was obtained for *pdhB* (Figure 1A). *B. subtilis* cells may utilize an additional acetyl-CoA producer for CshA acetylation, that is, acetyl-CoA synthetase encoded by *acsA* (Grundy et al., 1993). Thus, we tested whether a mutation of *acsA* affected the GI of *sigX-lacZ*. The strain bearing both *sigX-lacZ* and *acsA* showed GI of *sigX-lacZ*, indicating no involvement of *acsA* in the GI of *sigX-lacZ* in our condition, i.e., without acetate in the medium (right, Figure 1A). Furthermore, addition of acetate to the culture, which would enhance PDH-independent acetyl-CoA production, resulted in no induction of *sigX-lacZ* (left, Supplementary Figure S1A). This is a surprising observation, which has to be further explored.

Acetyl-phosphate could be the acetyl moiety source for CshA acetylation (Figure 1B). To investigate the influence of acetyl-phosphate on GI of *sigX-lacZ*, we examined the effects of both *ackA* and *pta* mutations, since both genes are involved in acetyl-phosphate production. GI of *sigX-lacZ* was observed in the both mutated cells, although it was moderately attenuated in the *ackA* strain for unknown reasons (Figure 1A). These suggest that acetyl-phosphate would not be involved in GI of *sigX*. Taken together, the results confirmed that acetyl-CoA produced by PDH is involved in acetylated CshA-dependent GI of *sigX*.

CshA is known to be associated with RNase J1 as well as RNase Y (Cascante-Estepa et al., 2016), and RNase Y is not involved in the GI of *sigX* (Ogura and Asai, 2016), and thus it is possible that CshA causes the GI of *sigX* through RNase J1. However, examination of *sigX-lacZ* expression in the strain bearing *sigX-lacZ* with the *rnjA* mutation revealed, however, that this is not the case (right, Supplementary Figure S1A).

### The YqfO and TsaD Genes Involved in Glucose Induction of *sigX-lacZ*

**FIGURE 2:**
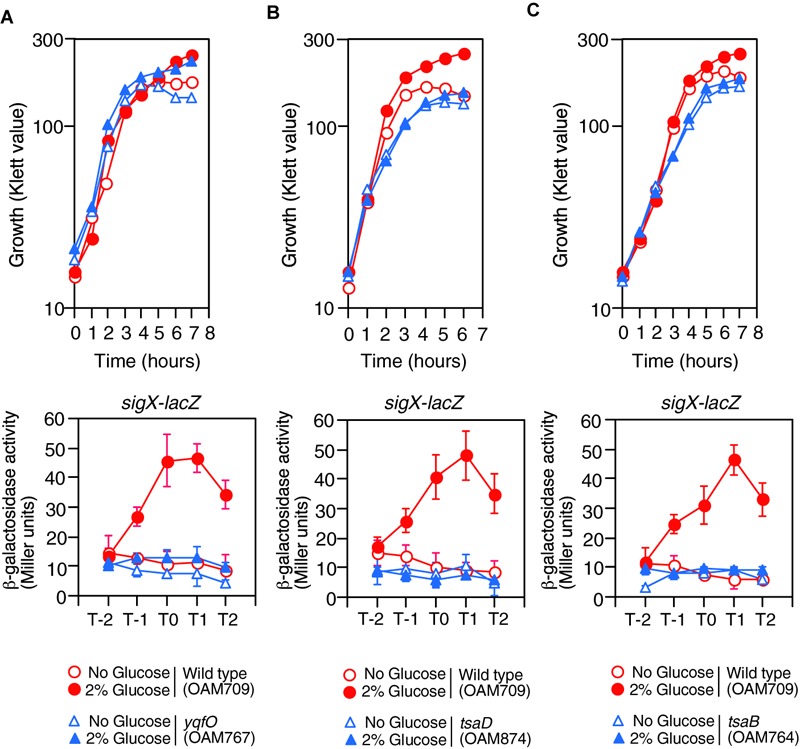
Growth and expression of *sigX-lacZ* in the mutants. **(A)** *yqfO*. **(B)** *tsaD*. **(C)** *tsaB*. The relevant genotypes are indicated below the panel. Growth curves of the wild-type and each mutant strain monitored by Klett colorimeter are shown (upper panel). Means of the β-Gal activities from three independent experiments and the standard deviations are shown (lower panel). The *x* axis is the same as in the legend to Figure 1.

Previously we performed transposon mutagenesis using *sigX-lacZ* (Ogura and Asai, 2016) and identified Tn-insertion into *trmK* and *tsaD*. Tn insertion into *trmK* encoding tRNA methyltranferase (Roovers et al., 2008) resulted in loss of GI of *sigX-lacZ* (left, Supplementary Figure S1B). To examine the effect of *trmK* disruption alone on GI, we used a *trmK* disruption mutant in which the expression of downstream *yqfO* encoding a conserved protein regulating gene transcription (Tascou et al., 2003) was ensured through an IPTG-inducible promoter. In the presence of IPTG, the strain showed reduced *sigX-lacZ* expression compared to that in the wild type, and thus GI was partially impaired (right, Supplementary Figure S1B). The observation suggested that *trmK* contributes to GI of *sigX* to some extent and the possible role of *trmK* will be analyzed elsewhere. When compared to the extent of impairment of GI between *trmK*::Tn and *trmK* with Pspac-*yqfO* mutants, a complete loss of GI was observed in the Tn-inserted *trmK* mutant. This suggested that Tn-insertion into *trmK* has a polar effect on downstream *yqfO*. We observed that without IPTG, similar expression pattern was observed, which suggested that leaky expression of the IPTG-dependent promoter would ensure the *yqfO* expression (data not shown). To solve the question, a disruption mutant of *yqfO* was constructed, and we examined the β-Gal activity of the *sigX-lacZ* strain with *yqfO*. The result showed that *yqfO* disruption caused elimination of GI (Figure 2A). Glucose addition to the wild-type strain showed enhancement of cell mass in the stationary phase, which was also observed in the *yqfO* mutant (Figure 2A). These indicate that it is disruption of *yqfO* that causes the elimination of GI.

The *tsaD* gene is located within a five-membered operon, of which the first gene is *thiL*, encoding a thiamine monophosphate kinase (Schyns et al., 2005). The product, thiamine pyrophosphate, is a cofactor for PDH. The following ORFs are *tsaE, tsaB, rimI* (ribosomal alanine *N*-acetyl transferase), and *tsaD*. We noted that *rimI* was not involved in CshA acetylation, because GI of *sigX-lacZ* was still observed in the *rimI* mutant (left, Supplementary Figure S1C). Without IPTG, similar expression pattern of *sigX-lacZ* was observed, suggesting leaky expression of the IPTG-dependent promoter would ensure the *tsaD* expression (data not shown). The *tsaD* gene encodes a component of enzyme complex, and the other components are encoded by *tsaB* and *tsaE* (Thiaville et al., 2015). With respect to *tsaD*, there is no possibility of a polar effect, as the *tsaD* ORF is located at the last position in the operon (Supplementary Figure S1C). TsaEBD is required for the synthesis of threonylcarbamoyl adenosine (t^6^A), which is used to modify tRNAs in bacteria. Disruption of *tsaD* resulted in decreased *sigX-lacZ* expression and loss of GI (Figure 2B). Irrespective of glucose addition, the *tsaD* mutant showed approximately one hour-delay of growth and we observed that glucose did not enhance cell mass at stationary phase (Figure 2B). We successfully disrupted *tsaB* by double crossover recombination. In the *tsaB* mutant, the GI of *sigX-lacZ* was also abolished and similar growth profiles were observed to those in the *tsaD* cells, as expected (Figure 2C); these are consistent with the TsaEBD requirement for GI.

**FIGURE 3:**
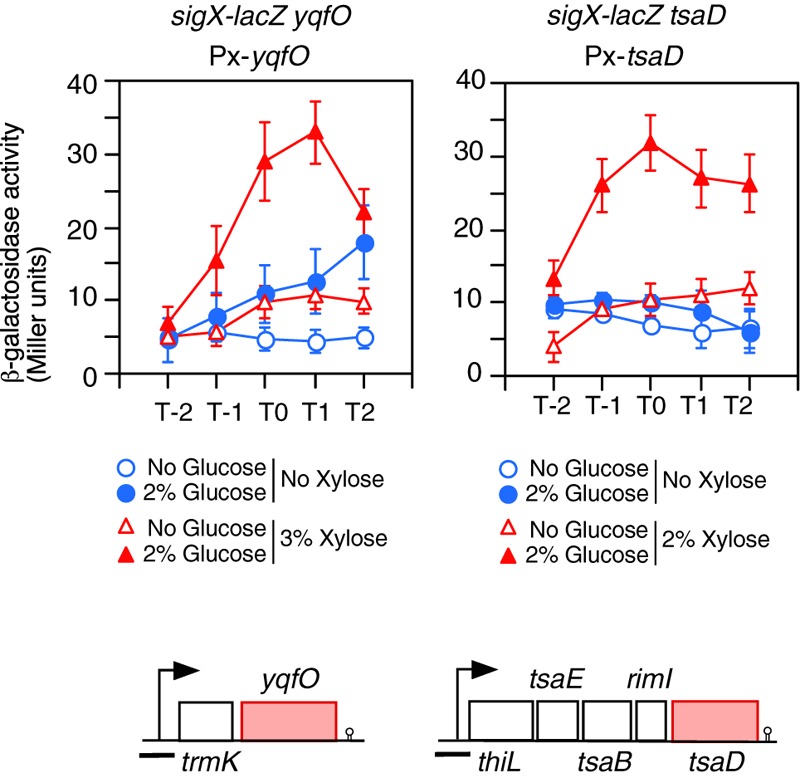
Complementation of the GI of *sigX-lacZ* in each gene disruption mutant by artificial induction of the gene. Means of the β-Gal activities from three independent experiments and the standard deviations are shown. The *x* axis is the same as in the legend to Figure 1. The relevant genotypes are indicated above the panel. Strains are as follows: left, OAM738; right, OAM739. The chromosomal structure of the operon containing the corresponding gene is shown under the panel. Boxes and bent arrows show open-reading frames and promoters, respectively. The gene names are shown along the boxes. The black bar shows the cloned promoter region for analyzing promoter expression.

Next, *yqfO* and *tsaD* strains with corresponding xylose-inducible genes at the *amyE* locus were constructed, and their β-Gal activities were examined. Without xylose, the *yqfO* strain showed some GI, probably due to leaky expression of the xylose-inducible promoter, whereas the *tsaD* strain showed no GI (Figure 3). In the presence of xylose, both strains showed significant GI of *sigX-lacZ* (Figure 3). These results indicate that *yqfO* and *tsaD* are involved in the GI of *sigX*. To understand whether these mutations influence the expression and GI of *sigX* independently or in the same regulatory cascade, we constructed a strain with disruption of both *tsaD* and *yqfO*. Then we examined the expression of *sigX-lacZ* in this double mutant. The expression and GI of *sigX-lacZ* in the double mutant were similar to the expression in either of the single mutants (right, Supplementary Figure S1C). Moreover, we tested the effect of the other double mutant, *ylxR* and *tsaD*, and obtained similar results (right, Supplementary Figure S1C). These results suggested that these genes might be in the same regulatory cascade. Subsequently we examined the possible relationship among these genes as described below.

### Positive Regulation of P*trm*K by YlxR

**FIGURE 4:**
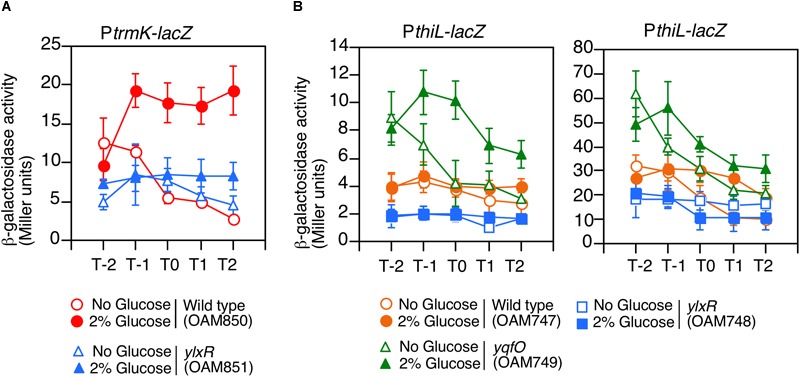
Expression of P*trmK-lacZ* and P*thiL-lacZ*. Means of the β-Gal activities from three independent experiments and the standard deviations are shown. The *x* axis is the same as in the legend to Figure 1. **(A)** P*trmK-lacZ*. **(B)** P*thiL-lacZ*. β-galactosidase activities measured using CRPG (right) instead of ONPG (left) as a substrate are shown.

YlxR is the NAP regulating many gene expression in early stationary phase (Ogura and Kanesaki, 2018), thus we examined the effect of the *ylxR* disruption on the expression of P*trmK*. As a result, we observed decreased P*trmK* expression in the *ylxR* disruption mutant (Figure 4A). Glucose addition resulted in the induction of P*trmK* in the wild type but not in the *ylxR* mutant. Based on these results, it was concluded that P*trmK* expression is positively regulated by *ylxR*.

### Regulation of P*thiL* by YlxR and YqfO

We examined the effects of disruption of *yqfO* and *ylxR* on the expression of P*thiL*, which drives *tsaD*. This was based on the assumption that there might be some regulatory relationship between the *yqfO, ylxR*, and *tsaD*. As a result, we observed that the expression of P*thiL* decreased moderately in the *ylxR* disruptant (left, Figure 4B). Thus, it was concluded that P*thiL* expression is positively regulated by *ylxR.* Moreover, P*thiL* expression was observed to increase and to be induced by the addition of glucose in the *yqfO* disruptant (left, Figure 4B). Therefore, *yqfO* plays negative regulatory role in P*thiL* expression and it was suggested that YqfO might repress GI of P*thiL*. The expression of P*thiL* was relatively low (around 4 Miller units), and thus, we used a highly sensitive substrate for β-galactosidase, CRPG. Then we observed similar results to those obtained by commonly used substrate, ONPG (right, Figure 4B). It should be noted that the decrease of P*thiL* expression was observed to a lesser extent in the *ylxR* strain when CRPG was used.

### *Cis-*Element for YlxR Revealed by Mutational Analysis of P*trm*K

**FIGURE 5:**
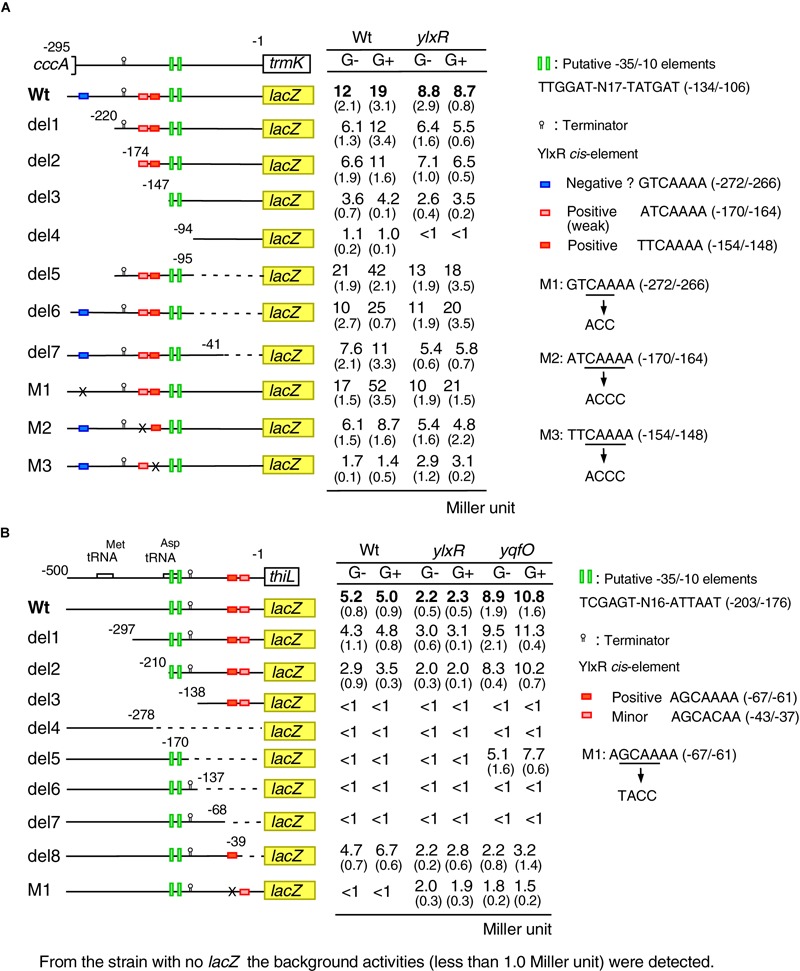
Expression of various P*trmK-lacZ* and P*thiL-lacZ* fusions. **(A)** P*trmK-lacZ*. **(B)** P*thiL-lacZ.* Strains were grown in sporulation medium with or without 2% glucose and sampled hourly from T-2 to T2. The averages and standard deviations of the observed peak values of β-Gal activities from the three independent cultures are shown. Numbers in parentheses show standard deviations. Structures of various *lacZ* fusions are depicted. Numbers indicate the nucleotide positions relative to the translation start nucleotide. The boxes on the line show the genes. The relevant genotype and the addition of glucose are shown (G- or G+). The construction of the mutated fusions is described in Supplementary Methods. Time-course data in the strains with wild-type fusion are shown in Figure 4.

We performed mutational analysis of the YlxR-dependent promoter, P*trmK*, using its *lacZ* fusions (Figure 5A). The time course data of the Wt fusion are shown in Figure 4A. We observed significant expression of del3 (-147/-1 relative to the translation start site) and del5 (-220/-95) but not del4 (-94/-1) both in the presence and absence of glucose, suggesting that the promoter is within the -147/-95 region carrying the putative promoter-like sequence TTGGAT-N17-TATGAT (-134/-106). These observations are consistent with those of previous genome-wide transcription analysis, where transcription initiation was observed immediately upstream of the *trmK* ORF, i.e., approximately 100 base pairs (Nicolas et al., 2012, subtiwiki). Decreased β-Gal activities in the *ylxR* disruptants in the presence of glucose were detected in the Wt (-295/-1), del1 (-220/-1) and del2 fusions (-174/-1) but not del3 (-147/-1), suggesting that the *cis-*element(s) for YlxR is within the -174/-147 region containing the candidate tandemly repeated sequences, ATCAAAA (-170/-164) and TTCAAAA (-154/-148). Indeed, introduction of nucleotide changes into the sequences resulted in dramatic loss of β-Gal activities in addition to loss of GI, indicating that these sequences are positive *cis-*elements for YlxR (M2 and M3). Notably, disruption of the downstream element had more profound effects compared to disruption of the upstream element. Next, we observed that in the wild-type background deletion of the -295/-220 region resulted in decreased and increased β-Gal activities between the Wt and del1 fusion pair and del5 and del6 pair, respectively. This may be because of the presence or absence of the downstream -95/-1 region in the fusions. Because the -295/-220 region contains a possible *cis-*element for YlxR, GTCAAAA (-272/-266), we introduced a nucleotide change into this sequence and examined its β-Gal activities (M1). As a result, the fusion showed greatly enhanced activities compared to the wild-type, demonstrating that this sequence would be a negative *cis-*element for YlxR. In the del5, del6, del7, M1, and M2 fusions but not in the M3 fusion, the disruption of *ylxR* resulted in the decreased fusion expression, compared to the expression of the corresponding fusion in the wild-type background, especially in the presence of glucose. This is consistent with the properties of these fusions carrying the positive *cis-*element for YlxR (-154/-148). We note that the expression of the del5, del6, and M1 fusions in the *ylxR* background was still induced by glucose at a lesser rate compared to that in the wild-type background. The cause of these observations remains unknown. Taken together, we detected positive two *cis-*elements for YlxR on the P*trmK* promoter.

### *Cis-*Element for YlxR Revealed by Mutational Analysis of P*thiL*

To clarify the possible *cis-*element for YlxR and YqfO on the P*thiL* promoter, we performed mutational analysis of this promoter (Figure 5B). The time course data of the Wt fusion are shown in Figure 4B. The –10 and –35 elements of P*thiL* have not been determined, but a putative terminator (stem-loop structure with U-tract) has been identified upstream of the *thiL* ORF (Rudner et al., 1993). To examine the location of transcription initiation activity in the upstream region of *thiL*, P*thiL-lacZ* fusions with various regions deleted were constructed, and their β-Gal activities were examined. The Wt (-500/-1 relative to the translation start site), del1 (-297/-1), and del2 (-210/-1) fusions showed β-Gal activity in the wild-type background, while the del3 (-138/-1) and del4 (-500/-278) fusions showed no β-Gal activity (Figure 5B). These results strongly suggested that the -35 and -10 elements for the *thiL* promoter are in the -210/-138 region. This region contains TCGAGT-N16-ATTAAT (-203/-176), the candidate -35 and -10 elements. It was observed that in the del8 (-500/-39) fusion a comparable expression was observed similar to that observed in the Wt fusion, while in the del5 (-500/-170), del6 (-500/-137), and del7 (-500/-68) fusions no expression was observed in the wild-type background. This suggested that there might be a positive *cis-*element for YlxR within the -68/-39 region, where there is no candidate promoter sequence. Importantly, this region contains similar *cis-*element for YlxR identified in P*trmK*, AGCAAAA (-67/-61). The introduction of nucleotide changes into this sequence resulted in the complete loss of β-Gal activity in the wild-type background (M1). These results suggested that the AGCAAAA (-67/-61) sequence serves as a positive *cis-*element for YlxR.

Next, we examined the expression of these mutant fusions in the *yqfO*-disruption background. We observed that *yqfO* disruption resulted in enhanced expression of the Wt fusion. Similarly, the disruption of *yqfO* enhanced the β-Gal activity in both del2 and del5 fusions, suggesting that *cis-*element for *yqfO* is in the core promoter region (-210/-170). However, the further construction of scanning mutants within the region required to identify the *cis-*element is beyond the scope of this study. The del6 and del7 fusions with the promoter in addition to the terminator showed no β-Gal activity even in the *yqfO* background, although del5 expression was observed in the *yqfO* strain, suggesting that *yqfO* disruption could increase the expression due to the lack of the terminator. It should be noted that in the *yqfO* background, del8 fusion lacking *cis-*element like sequence AGCACAA (-43/-37) showed no enhancement of β-Gal activity. This suggested that this sequence may be required for enhancement by disruption of *yqfO*, the exact role of this sequence, however, remains unclear.

In the Wt, del1, del2, and del8 fusions with the *cis-*element for YlxR, *ylxR* disruption decreased the expression in both the presence and absence of glucose to various extents. This is consistent with the positive role of YlxR in P*thiL* expression. We note that *ylxR* disruption did not cause complete loss of fusion expression in the Wt fusion, suggesting a secondary and weak positive effect on P*thiL* expression by the *ylxR* disruption. In fact, M1 showed low levels of expression in the *ylxR* disruptant. This may be due to the negative effect of *ylxR* disruption on *yqfO* expression (Figure 4A), leading to enhancement of P*thiL* expression.

### YlxR-Binding to P*thiL* and P*trmK*

YlxR was first identified as a non-specific DNA-binding protein, but it may contain a preferential binding site in the nucleotide sequence as well as other NAPs (Sandhya et al., 2015; Ogura and Kanesaki, 2018). As expected, YlxR bound to the DNA probes with P*trmK* and P*thiL* in the EMSA (Supplementary Figure S2). To examine preferential binding of YlxR to the identified the three putative *cis-*elements, we performed EMSA using the wild-type and mutated P*trmK* probes for YlxR. The probe contains three *cis-*elements for YlxR. When M1 and M3 were independently introduced into the probe, similar DNA-binding affinity of YlxR was observed, although the retardation of the YlxR-DNA complex was slightly lowered, indicating lower molecular mass of the complex (compare lane 4 to lane 12, left, Supplementary Figure S2A), or affinity of the probe to YlxR was lowered (compare lane 2 to lane 6, left, Supplementary Figure S2A). Next, we used the probe with both M1/M3 mutations (right, Supplementary Figure S2A). In lane 2 of Supplementary Figure S2A, a sharp band corresponding to the probable YlxR/wild-type DNA complex was observed, indicating that 0.1 μM was critical concentration of YlxR, where DNA-binding was partially observed. On the other hand, no band was observed for the M1/M3 probe at 0.1 μM YlxR (lane 7). Moreover, when lane 5 and lane 10 were compared, all probes were retarded due to DNA-binding of YlxR. However, slightly lowered mobility was observed for the mutant probe. These show the differential binding of YlxR to the wild-type and mutated probes. Finally, we tested whether YlxR binds preferentially the *cis-*element in P*thiL*. When lane 3 and lane 7 were compared, YlxR showed weak DNA-binding activities to the probe with a mutated *cis-*element compared to the wild-type probe (Supplementary Figure S2B). These results suggest that YlxR preferentially binds to its *cis-*elements, although the difference in affinity between preferential binding sites and non-specific ones was small.

### Transcription of P*pdhABCD*

**FIGURE 6:**
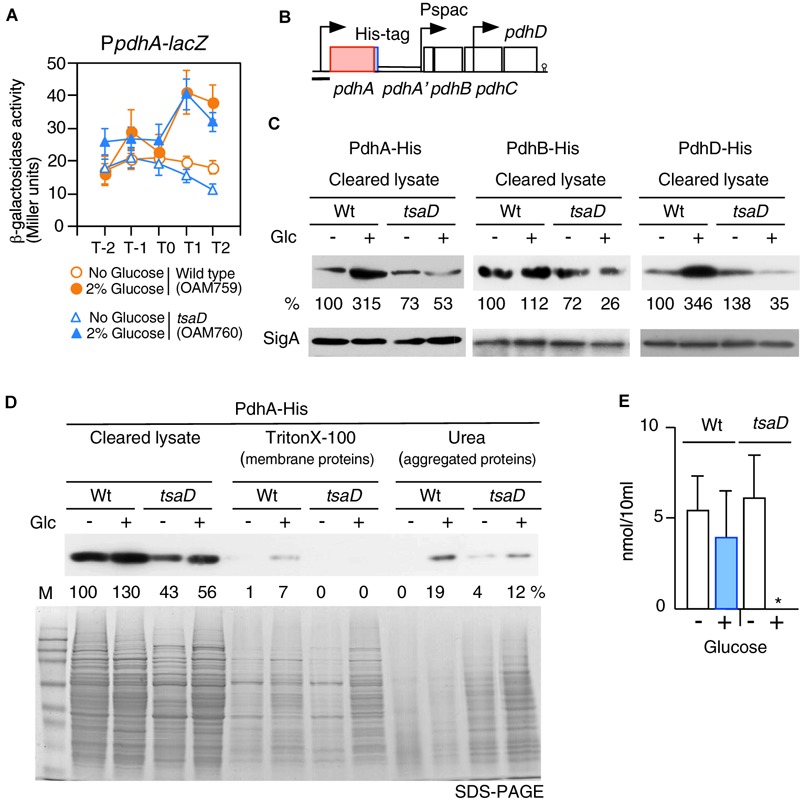
Expression of P*pdhA-lacZ* and measurement of soluble PDH subunits in the *tsaD* mutant. **(A)** Expression of P*pdhA-lacZ.* Means of the β-Gal activities from three independent experiments and the standard deviations are shown. The *x* axis is the same as in the legend to Figure 1. **(B)**. The chromosomal structure of the strain with *pdhA*-*His* (OAM761) is shown. The legend for graphics is the same as that of Figure 3. The black bar shows the cloned promoter region for analyzing promoter expression. The chromosomal structures of the strains bearing PdhB-His and PdhD-His are similar to that of OAM761 (not shown). **(C)** Western analysis of PdhA-His, PdhB-His and PdhD-His. For the culture of each strain bearing His-tagged Pdh subunit [PdhA-His, wild type (OAM761), *tsaD* (OAM762); PdhB-His, wild type (OAM777), *tsaD* (OAM778)], 1 mM IPTG was added in sporulation medium with or without 2% glucose (“Glc” means glucose addition). For the cultures of strains with PdhD-His, wild type (OAM779) and *tsaD* (OAM780), no IPTG was added. Equal protein amounts of soluble fractions were analyzed in western blot using anti-His-tag monoclonal antibody for detection of each His-tagged protein. As a control, SigA detected by anti-SigA antibody is shown. **(D)** Western blot analysis of cleared cell lysates, “membrane protein” fractions (Triton X-100 extracts), and “aggregated protein” fractions (insoluble fractions by Triton × -100, solubilized by urea-containing buffer) from the wild type and *tsaD* cells for PdhA-His. OAM761 and OAM762 cells were cultivated and treated as described in the Materials and Methods. Cell mass equivalents were analyzed using both western blot and SDS-PAGE stained by Coomassie. “M” indicates molecular weights marker (Amersham full range rainbow marker). **(E)** Examination of cellular acetyl-CoA pool. Samples from two independent cultures (wild type, 168; *tsaD*, OAM734) were analyzed three times, and standard deviations are shown. ^∗^indicates that the value is less than the detection limit (0.5 nmol/10 ml).

TsaD is a component of TsaEBD required for the synthesis of t^6^A-modified tRNA; thus TsaD might be involved in translational control of several proteins. As an acetyl-CoA producer, PDHc is required for CshA acetylation (Figure 1B). Subunits of PDHc are known to be most actively synthesized proteins in the growing *B. subtilis* cells (Eymann et al., 2004), suggesting that PDHc synthesis would be under transcriptional, translational and post-translational control. Therefore, it is possible that subunits of PDHc are under translational or post-translational control involving TsaD. First, we examined the transcription of the *pdhABCD* operon. A previous study showed that this operon transcription increases in the presence of glucose (Blencke et al., 2003). We confirmed this observation using P*pdhA-lacZ* (Figure 6A). Furthermore, we observed that this increase is not dependent of TsaD, YlxR, CcpA, CcpC, CcpN, or CggR (Figure 6A, data not shown). These results suggest an unknown mechanism for the GI of P*pdhA*. The *pdhABCD* operon has an internal promoter (Nicolas et al., 2012). To test possible effects of the *tsaD* mutation on the internal promoter, *pdhD-lacZ* was constructed (Supplementary Figure S3). The expression and GI of *pdhD-lacZ* were similar in both wild-type and *tsaD* cells. Thus, it was confirmed that the *tsaD* disruption also plays no role in the GI of *pdhD-lacZ*.

### Decrease of PDHc Subunits in the *tsaD* Disruptant With Glucose

To test the influence of *tsaD* disruption on PdhA at the protein level, western analysis of PdhA was performed. For the detection of PdhA, a His-tag was genetically introduced into the C-terminal end of *pdhA* (Figure 6B). To sustain downstream *pdhBCD* transcription, IPTG was added to the culture of the *pdhA*-*His* strain. The strain grew normally, suggesting no harmful influence of the His-tag addition to PdhA (data not shown). After cells were lysed, soluble fractions were obtained by centrifugation. Western analysis of soluble fractions with equal protein amounts of the wild-type strain using anti-His-tag antibody confirmed that glucose addition increased PdhA-His at the protein level (Figure 6C). A similar western analysis of *tsaD* cells revealed that glucose addition significantly decreased the amount of soluble PdhA-His, compared to that of the wild-type strain with glucose, even though transcription was similarly enhanced (Figure 6). This suggested some post-transcriptional defects at the protein level.

During preparation of the cleared cell lysate, the insoluble fractions contained membrane- and aggregated-proteins. Thus, we separated these insoluble fractions to “membrane protein” and “aggregated protein” fractions. Notably, each fraction was derived from the equal cell amounts, which is different from the method used for the results shown in Figure 6C. These fractions were analyzed by western blot to detect PdhA-His (Figure 6D). With this method, however, we also obtained similar results in soluble fractions to those shown in Figure 6C. Despite the presence of PdhA-His in the “membrane protein” fraction, we wanted to verify its localization, since PdhA-YFP has previously been identified in the cytoplasm (Monahan et al., 2014). We similarly observed localization of PdhA-GFP in the cytoplasm (Supplementary Figure S4), which refutes evidence for membrane localization of PdhA. Thus, this detection may be due to the contaminated cytosolic PdhA because of cellular abundance. In the “aggregated protein” fraction from wild type cells with glucose, PdhA-His were detected. Perhaps an abundance of *pdh* mRNA in the cell creates a burden for the translational machinery, leading to generation of aggregated PdhA-His. Overexpression of protein has often caused protein aggregation (Bednarska et al., 2013).

From *tsaD* cells with and without glucose, significant and minor levels of PdhA-His, respectively, were detected in the urea fractions (Figure 6D). These results showed that in the *tsaD* cells, misfolded or aggregated PdhA was generated. We noted using SDS-PAGE that the “aggregated protein” fraction was enriched in the *tsaD* cells, suggesting that the *tsaD* mutation has a global effect on protein quality control (Figure 6D). In the “membrane proteins” fraction from the *tsaD* cells with glucose, slightly more amount of proteins was observed compared to that without glucose. The detected proteins initially aggregated or misfolded and thus were fractioned into the insoluble pellets, which were solubilized by a weak detergent like Triton X-100. This experiment was further performed five times, with three trials yielding similar results to that shown in Figure 6D. In two cases, PdhA-His was scarcely detected in the urea fractions of wild type or *tsaD* cells (data not shown). Although it is not well understood how the fate of misfolded proteins is determined (i.e., refolding, amyloid formation, or degradation), aggregates are considered pathway intermediates (Supplementary Figure S5; Bednarska et al., 2013; Balchin et al., 2016). Thus, in the former cases, relatively small amounts of aggregated PdhA-His in the *tsaD* cells could be detected because of protein aggregation. In contrast, in the latter cases most of insoluble PdhA-His could be degraded through the aggregation state. Taken together, these results strongly suggest that protein quality control of PdhA requires TsaD especially in the glucose-added condition.

There remained the possibility that TsaD is needed for the protein quality control of the other PDHc components. To examine this, similar western analyses were performed; however, the antigenic activity of PdhC-His was too low, and thus no information was obtained (data not shown). Western analysis of soluble PdhB-His and PdhD-His showed significant decreases in the *tsaD* cells with glucose like PdhA-His (Figure 6C). These findings also strongly suggested that protein quality control of PdhB and PdhD requires TsaD in the glucose-added condition. In western analysis of insoluble fractions His-tagged Pdh proteins were scarcely detected (data not shown). These results suggested that probable protein aggregates from PdhB-His and PdhD-His might be susceptible to protein degradation.

### Acetyl-CoA Pool in *tsaD* Cells

When glucose was added to culture of the *tsaD* disruption mutant, levels of soluble PDHc subunits were severely decreased. Thus, the activity of PDHc may be inhibited by the decrease of protein amount as well as disturbances in the stoichiometry of PDHc in the mutant. To examine this, intracellular acetyl-CoA concentrations were determined (Figure 6E). Glucose addition increased PDHc in wild-type cells, but the size of the acetyl-CoA pool remained similar. There was also a similar level of acetyl-CoA observed in *tsaD* cells without glucose. In contrast, in *tsaD* cells with glucose, a severe decrease in the levels of acetyl-CoA was observed. This should decrease the acetylation of CshA in the presence of glucose, leading to the elimination of the GI of *sigX/M*.

## Discussion

**FIGURE 7:**
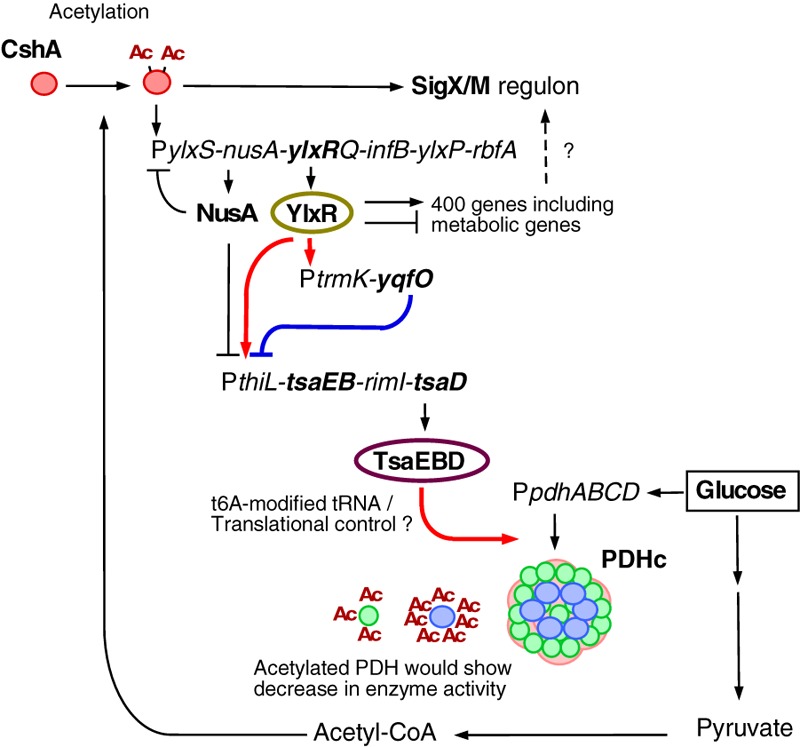
Suggested model of glucose-responsive system (GRS). The thick lines show the regulatory pathways identified and analyzed in this paper. The other pathways have been analyzed in Ogura and Asai, 2016 (acetylation of CshA and *sigX/M* regulation), Ogura and Kanesaki, 2018 (CshA-dependent regulation of P*ylxS* driving expression of YlxR, which regulates metabolic genes), and Mondal et al., 2016 (NusA inhibits P*ylxS* and P*thiL*). With respect to PDHc, this cartoon does not correctly reflect the actual structure (Zhou et al., 2001). Green circle, PdhE1 (*pdhA* and *pdhB*); blue circle, PdhE3 (*pdhD*); pink moiety, PdhE2 core (*pdhC*). PDHc is also known to be acetylated (under glucose-rich conditions, at 3 moieties of PdhA and 7 moieties of PdhD, Kosono et al., 2015), and acetylation reduces enzyme activity. Arrows indicate transcription, translation, acetylation, enzyme reaction, protein association or transcriptional activation. T-bars indicate transcriptional repression or attenuation. Ac, acetyl moiety; PDHc, pyruvate dehydrogenase complex; RNAP, RNA polymerase.

Based on the data obtained in this study, we suggest a new glucose-responsive system (GRS) that includes protein lysine acetylation, transcriptional regulation, and protein quality control. We present a model for the regulation of the acetylation of CshA, which stimulates the formation of sigma X/M-bound RNAP (Figure 1B, 7).

YqfO is a conserved protein among Firmicutes and bears a nif3 conserved domain with a suggested function related to transcriptional regulation (Tascou et al., 2003). The structure of the *Bacillus cereus* YqfO has been resolved and suggests that YqfO may have a ligand-binding domain (Godsey et al., 2007). YqfO was demonstrated to be involved in P*thiL* expression, and thus probably *yqfO* disruption may have impaired the regulation of *tsaEDB*, leading to loss of GI of *sigX* through the dysregulation of PDHc expression. However, YqfO may be indirectly involved in regulation of GI of *sigX*.

YlxR is also involved in the expression of *thiL*. According to this model, the acetylation of CshA is a self-reinforcing system. The disruptions of *ylxR* and *yqfO* result in the elimination of the GI of *sigX/M*. This is due to the disruption of robust and fine-tuned expression of the *tsaEBD*-containing operon, the products of which play a role in the quality control of PDHc subunits. However, in the earlier genome-wide analysis using *ylxR* cells in the presence of glucose the *thiL* operon was not detected as a target for YlxR (Ogura and Kanesaki, 2018). This seems to be an inconsistent result according to the *lacZ* analysis. These are inherent biases in this type of genome-wide analysis, which may be resulting from specific mRNA degradation during the sample preparation. Thus, the results in this study are plausible.

According to the expression landscape data (Nicolas et al., 2012, subtiwiki), under some conditions transcription initiation of the *thiL* operon was observed immediately upstream of the *thiL* gene; however, the more upstream region was not analyzed because this region contains rRNA operons. On the other hand, the P*thiL* promoter is known to be one of the target promoters for the NusA-dependent termination/attenuation system. Genome-wide analysis of the effects of NusA-depletion revealed enhanced expression of the *thiL* operon, indicating that NusA serves as a negative regulator for P*thiL* (Mondal et al., 2016). The detected promoter in our analysis associated with its downstream terminator is likely involved in NusA-dependent regulation. Taken together, it is likely that the proposed *thiL* promoter is functional.

One of the outputs of this newly suggested GRS is GI of *sigX/M*, which shows wider roles of SigX/M beyond the nature of ECF sigma factor in surface stress response. In other words, expression of the SigX/M regulons responds to nutritional changes. This system would regulate the cellular acetyl-CoA pool, which is one of the sources for protein lysine acetylation, through PDHc under glucose-rich growth conditions. PDHc would play a critical role in CshA acetylation as a supplier of acetyl-CoA. PdhA and PdhD were acetylated under glucose-rich conditions in *B. subtilis* (Kosono et al., 2015). Another study reported that all components of PDHc were acetylated (Carabetta et al., 2016). The transcription of *pdhABCD* is stimulated by glucose addition, leading to larger amounts of PDH, while we observed similar levels of intracellular acetyl-CoA. In many bacteria including *Salmonella*, the acetylation of many enzymes for glycolysis has been reported to reduce their enzymatic activities (Wang et al., 2010; Nakayasu et al., 2017), and in *B. subtilis*, PdhC and PdhD activities were controlled at the protein modification or activity level but not at the enzyme concentration level (Chubukov et al., 2013). Considering these data, *B. subtilis* PDHc activity may be down regulated by its acetylation.

We found that TsaD required for the synthesis of t^6^A in tRNA plays some role in the protein quality control of PDHc subunits in the presence of glucose. Under such a condition, transcription of mRNA encoding Pdh subunits is enhanced, which could burden the translational machinery without t^6^A-modified tRNA. t^6^A is located at position 37 of the anticodon loop in tRNAs that decode ANN codons. t^6^A is universally conserved across the three domains of life (Thiaville et al., 2014). In the most bacterial genomes sequenced to date, homologs of TsaE, TsaB, and TsaD have been found. In many bacteria, these genes are essential (Thiaville et al., 2014, 2015). However, in *Deinococcus radiodurans* R1, *tsaB* and *tsaD* are non-essential, and in *Synechocystis* sp. PCC6308 *tsaD* is non-essential. In both bacteria, the essentiality of the rest of the genes in TsaEBD has not been experimentally determined. All three genes were reported to be essential in *B. subtilis*, as initial attempts for constructing these gene disruptions were not successful (Kobayashi et al., 2003). Later two groups have reported that *tsaE* is non-essential (Hunt et al., 2006; Koo et al., 2017). Tn insertion into *tsaD* was confirmed by PCR analysis and *tsaB* was able to be disrupted in this study, demonstrating that these genes were not essential. However, it should be noted that some unknown suppressor mutation for lethality within the genome could help the generation of the *tsaD* or *tsaB* disruptant, though there were no observations such as low transformation rate during gene disruption or transfer process to suggest suppressor mutation.

In the depletion or disruption mutants of the genes involved in the synthesis of t^6^A, various phenotypes have been observed, such as those about transcriptional control, cell division, and cyanophycin accumulation (Zuther et al., 1998; Lei et al., 2012; Bitoun et al., 2014). The mechanism by which these phenotypes are caused by TsaEBD is currently unknown. In the *B. subtilis tsaD* cells with glucose, the intracellular acetyl-CoA pool was not detected. This observation does not indicate that the *tsaD* cells lack acetyl-CoA, because the *tsaD* cells was able to grow with about one-h delay compared to growth of wild-type cells. Perhaps smaller amount of Pdh components impaired acetyl-CoA pool in the *tsaD* cells with glucose, but active acetyl-CoA flux may function to some extent. In the *E. coli tsaD*-depleted mutant, a reduction in assembled PDHc was observed and this was attributed to the accumulation of glycated components of PDHc (Katz et al., 2010). Protein glycation is thought to eventually produce toxic compounds and the authors pointed out that TsaD is a possible glycopeptidase for glycated PDHc. However, the archaeal TsaD homolog Kae1 showed no protease activity (Hecker et al., 2007), and there are no other reports for the protease activity of TsaD. Thus, the speculation that removal of glycated PDHc by TsaD in *B. subtilis* may contribute to quality control of PDHc is not likely. Moreover, the depletion or deficiency of t^6^A synthesis should affect translation, although the direct protein target of t^6^A-modified tRNA is unknown in bacteria (Thiaville et al., 2015). Recently, the idea has emerged that translational speed is evolutionarily optimized for folding of each protein (Balchin et al., 2016). In yeast and nematoda, the lack of anticodon loop modifications in tRNA results in ribosome pausing and slower translation rate, leading to the misfolding of proteins (Patil et al., 2012; Nedialkova and Leidel, 2015). Since ribosome surface is associated with the chaperons for nascent polypeptide folding, leading to cotranslational protein-folding, operon-coded proteins may be concomitantly susceptible to protein misfolding (Balchin et al., 2016). Thus, it is reasonable that PDHc subunits showed similar responses in the *tsaD* cells with glucose as to the protein quality control. Our finding that a deficiency of t^6^A synthesis in *tsaD* cells resulted in decreased soluble PDHc subunits in *B. subtilis* is consistent with these former observations. This discussion premised the direct translational control of PDHc by the TsaEBD complex. However, there are the other possibilities, including impaired global protein quality control indirectly resulting in the decreased PDHc.

The suggestion of this GRS revealed that the cellular glucose response was not completely clarified in previous studies. Additional detailed studies of this system will provide insight into the physiology of bacteria in adapting to glucose-rich conditions.

## Author Contributions

MO designed the study, contributed to acquisition, analysis and interpretation of the data, and wrote the manuscript. TS and KA contributed to the acquisition of the data.

## Conflict of Interest Statement

The authors declare that the research was conducted in the absence of any commercial or financial relationships that could be construed as a potential conflict of interest.
