# Supplementary material for: Bacillus subtilis YlxR, Which Is Involved in Glucose-Responsive Metabolic Changes, Regulates Expression of tsaD for Protein Quality Control of Pyruvate Dehydrogenase
Source: Front Microbiol. 2019 May 1;10:923. doi: 10.3389/fmicb.2019.00923 (PMC6504816; doi:10.3389/fmicb.2019.00923)
Supplement: Supplementary file 1 [file Data_Sheet_1.PDF]

Bacillus subtilis YlxR, which is involved in glucose-responsive metabolic changes, regulates expression of *tsaD* for protein quality control of pyruvate dehydrogenase

Mitsuo Ogura, Tsutomu Sato and Kimihiro Abe

Supplementary methods

Strain construction

The *tsaB*::Tc^r units were constructed using PCR. Briefly the upstream and downstream regions of the concerned genes and Tc^r from pBEST304 (Itaya, 1992) were amplified using the indicated primers (Table S2) and then combined by PCR. These units were directly used for transformation of *B. subtilis* 168. From the resultant Tc^r strain total DNA was taken. That was used in PCR to confirm the expected chromosomal structure as template.

Plasmid construction

To construct pDH88-ykqC, the PCR product was amplified by using the oligonucleotide pair, YkqC-3/YkqC-4, digested with HindIII/XbaI, and cloned into pDH88 treated with the same enzymes (Henner, 1990). To construct pIS-trmK-del1, -del2, -del3, -del4, del5, -del6, -del7, and -M1, the PCR products were amplified by using the oligonucleotide pairs, pIS-trmK-E-del1/pIS-trmK-B, pIS-trmK-E-del1.1/pIS-trmK-B, pIS-trmK-E-del2/pIS-trmK-B, pIS-trmK-E-del3/pIS-trmK-B, pIS-trmK-E-del1/pIS-trmK-B-del1, yqfO-Eco/pIS-trmK-B-del1, yqfO-Eco/pIS-trmK-B-del1.1, and yqfO-M6/pIS-trmK-B, digested with EcoRI/BamHI, and cloned into pIS248 treated with the same enzymes (Tsukahara and Ogura, 2008). To construct pIS-trmK-M2 and pIS-trmK-M3, the PCR-based introduction of the mutation was adopted using oligonucleotides yqfO-Eco/pIS-trmK-B/pIS-trmK-M1a/pIS-trmK-M1b and yqfO-Eco/pIS-trmK-B/pIS-trmK-M2a/pIS-trmK-M2b as described previously (Ogura and Tanaka, 1996). The PCR products were digested with EcoRI/BamHI, and cloned into pIS248 treated with the same enzymes (Tsukahara and Ogura, 2008). To construct pDG1729-thiL-del2, -del4, -del5, -del6, del7 and -del8, the PCR products were amplified by using the oligonucleotide pairs, pDG1729-gcp-E2.53/pDG1729-gcp-B, pDG1729-gcp-E/gcp-R2, pDG1729-gcp-E/pDG1729-gcp-B2, pDG1729-gcp-E/pDG1729-gcp-B3, pDG1729-gcp-E/pDG1729-gcp-B4, and pDG1729-gcp-E/pDG1729-gcp-B5, digested with EcoRI/BamHI, and cloned into pDG1729 treated with the same enzymes (Guérout-Fleury et al., 1996). To construct pDG1663-thiL-del1 and -del3, the PCR products were amplified by using the oligonucleotide pairs pDG1729-gcp-E2/pDG1729-gcp-B and pDG1729-gcp-E3/pDG1729-gcp-B, digested with EcoRI/BamHI, and cloned into pDG1663 treated with the same enzymes (Guérout-Fleury et al., 1996). To construct pDG1729-thiL-M1,

the PCR-based introduction of the mutation was adopted using oligonucleotides pDG1729-gcp-E/pDG1729-thiL-M1/pDG1729-thiL-M2/pDG1729-gcp-B as described previously (Ogura and Tanaka, 1996). The PCR product was digested with EcoRI/BamHI, and cloned into pDG1729 treated with the same enzymes (Guérout-Fleury *et al*., 1996). To construct pMUTIN-pdhD, the PCR product was amplified by using the oligonucleotide pair, pMUT-pdhD-E/pMUT-pdhD-B, digested with EcoRI/BamHI, and cloned into pMUTIN treated with the same enzymes (Vagner et al., 1998). To construct pSG1154-pdhA, the PCR product was amplified by using the oligonucleotide pair, pSG1154-pdhA-Xh/pSG1154-pdhA-Ec, digested with XhoI/EcoRI, and cloned into pSG1154 treated with the same enzymes (Lewis and Marston, 1999).

Electromobility shift assay (EMSA)

Methods for purification of YlxR was described previously (Ogura and Kanesaki, 2018). The oligonucleotides used for preparing probes are shown in Table S2. YlxR was incubated with each DNA probe (20 fmol) in 20 μl of a buffer containing 10 mM Tris-HCl (pH 8.0), 100 mM KCl, 10 mM $MgCl_2$, 1 mM DTT, and 1 mM EDTA for 15 min at 25°C. 1 μg of poly(dI-dC) (Amersham) was added to the reaction. Procedure of electrophoresis and detection of DNA was described previously (Ogura et al., 2014).

Microscopic observations

The methods were performed as described previously (Ogura, 2016). Microscopy was performed with an Olympus BX50 phase-contrast and fluorescence microscope with a 100× UplanApo objective (Olympus, Tokyo, Japan). Images were captured using a CoolSnap HQ charge-coupled device camera (Photometrix, AZ, USA) and Metamorph v7.6.5.0 software (Universal Imaging, PA, USA). GFP fluorescence was visualized using a NIBA filter set (Olympus).

References to Supplementary Methods

Balchin, D., Hayer-Hartl, M., and Hartl, F.U. (2016) In vivo aspects of protein folding and quality control. S*cience* **353**: aac4354.

Guérout-Fleury, A.M., Frandsen, N., and Stragier, P. (1996) Plasmids for ectopic

integration in *Bacillus subtilis*. *Gene* **180**: 57-61.

Henner, D.J. (1990) Inducible expression of regulatory genes in *Bacillus subtilis*. *Methods Enzymol* **185:** 223-228.

Itaya, M. (1992) Construction of a novel tetracycline resistance gene cassette useful as a marker on the *Bacillus subtilis* chromosome. *Biosci Biotechnol Biochem* **56**: 685-686.

Lewis, P.J., and Marston, A.L. (1999) GFP vectors for controlled expression and dual labelling of protein fusions in *Bacillus subtilis. Gene* **227**:101-110.

Ogura, M. (2016) Post-transcriptionally generated cell heterogeneity regulates biofilm formation in *Bacillus subtilis*. *Genes Cells* **21**: 335-349.

Ogura, M., and Tanaka, T. (1996) Transcription of *Bacillus subtilis degR* is σ^D-dependent and suppressed by multicopy *proB* through σ^D. *J Bacteriol* **178**: 216-222.

Ogura, M., and Kanesaki, Y. (2018). Newly identified nucleoid-associated-like protein YlxR regulates metabolic gene expression in *Bacillus subtilis*. *mSphere* 3, e000501-18.

Ogura, M., Yoshikawa, H., and Chibazakura, T. (2014). Regulation of the Response Regulator Gene *degU* through the Binding of SinR/SlrR and Exclusion of SinR/SlrR by DegU in *Bacillus subtilis*. *J. Bacteriol.* 196, 873-881.

Tsukahara, K., and Ogura, M. (2008) Promoter selectivity of the *Bacillus subtilis* response regulator DegU, a positive regulator of the *fla/che* operon and *sacB*. *BMC Microbiol* **8**: 8.

Vagner, V., Dervyn, E., *and* Ehrlich, S.D. (1998) A vector for systematic gene inactivation in *Bacillus subtilis*. *Microbiology* **144**: 3097–3104.

Legends to Figures

Fig. S1. Effects of various mutations on GI of *sigX-lacZ*. Cells were grown in sporulation medium with or without 2% glucose and sampled hourly. 30 mM acetate was added instead of glucose and then pH was adjusted to 7.0. Means of β-Gal activities shown in Miller units from three independent experiments and the standard deviations are shown. The X-axis represents the growth time in hours relative to the end of vegetative growth (T0). All strains bear *sigX-lacZ* at *thrC* and the relevant genotypes are indicated below the panel. For the experiment of the strains bearing *trmK* and *rimI*, 1mM IPTG was added. The chromosomal structures of the *trmK* and *rimI* mutants are shown. Bent arrows and boxes show promoters and ORFs, respectively. The double line indicates the inserted vector sequence.

Fig. S2. EMSA using YlxR. Each sample was analyzed via electrophoresis in a 6% non-denaturing polyacrylamide gel. The numbers associated with the probe name indicate the spanned promoter region. C and F show protein-DNA complex and free probe, respectively. A. The (Wt and M1), and (M3 and [M1, M3]) probes were prepared by PCR using oligonucleotide pairs, yqfO-EMSA1/yqfO-F-bio and yqfO-EMSA1/pIS-trmK-M2b, respectively. The (Wt and M3), and (M1 and [M1, M3]) were amplified from pIS-trmK and pIS-trmK-M1, respectively. B. The Wt and M1 probes were prepared by PCR using oligonucleotide pairs, thiL-F4/gcp-biotin-R and thiL-F4M/gcp-biotin-R, respectively.

Fig. S3. Expression of *pdhD-lacZ*. Cells were grown in sporulation medium with or without 2% glucose and sampled hourly. Means of β-Gal activities shown in Miller units from three independent experiments and the standard deviations are shown. The X-axis represents the growth time in hours relative to the end of vegetative growth (T0). The relevant genotypes are indicated below the panel. The chromosomal structure of the *pdhD-lacZ* fusion is shown. Bent arrows and boxes show promoters and ORFs, respectively. The double line indicates the inserted vector sequence.

Fig. S4. Localization of PdhA-GFP. OAM783 (Wt) and OAM814 (*tsaD*) cells were grown in 50 ml of sporulation medium with or without 2% glucose. Next, 100 microL of the culture at T0 was centrifuged, and the cells were resuspended in 10 microL of sporulation medium. Portions (2 microL) of each sample were mounted on poly-L-

lysine pre-coated glass slides. PC, Phase contrast. PdhA-GFP is expressed from its own promoter.

Fig. S5. Schematic representation of fate of misfolded proteins. This is based on Balchin et al., 2016.

Table S1. Strains and plasmids used only in the Supplementary section of the study and Fig. 4.

Strain	Genotype	Reference or source
OAM763	*trpC2 thrC*::*sigX*-*lacZ* Wt (-43 to +262[1], Em^r) Pspac-*rnjA* (Cm^r)	This study
OAM875	*trpC2 thrC*::*sigX*-*lacZ* Wt (-43 to +262, Em^r) *trmK*::Tn (Km^r)	This study
YqfNd	*trpC2 yqfN (trmK)* (Em^r *lacZ*)	BSORF
YdiDd	*trpC2 ydiD (rimI)* (Em^r *lacZ*)	BSORF
OAM766	*trpC2 thrC*::*sigX*-*lacZ* Wt (-43 to +262, Em^r) *trmK* (Em^r *lacZ*::Tc^r)	This study
OAM876	*trpC2 thrC*::*sigX*-*lacZ* Wt (-43 to +262, Em^r) *rimI* (Em^r *lacZ*::Tc^r)	This study
OAM877	*trpC2 thrC*::*sigX*-*lacZ* (Em^r) *yqfO* (Em^r *lacZ*::Tc^r) *tsaD*::Tn (Km^r)	This study
OAM878	*trpC2 thrC*::*sigX*-*lacZ* (Em^r) *ylxR* (Em^r *lacZ*::Tc^r) *tsaD*::Tn (Km^r)	This study
OAM775	*trpC2 pdhD*-*lacZ* (Em^r)	This study
OAM776	*trpC2 pdhD*-*lacZ* (Em^r) *tsaD*::Tn (Km^r)	This study
OAM783	*trpC2 amyE*::Xyl-PpdhA-*pdhA*-*gfp* (Sp^r)	This study
OAM814	*trpC2 amyE*::Xyl-PpdhA-*pdhA*-*gfp* (Sp^r) *tsaD*::Tn (Km^r)	This study
OAM852	*trpC2 amyE*::P*trmK*-*lacZ* (del1 Cm^r)	This study
OAM853	*trpC2 amyE*::P*trmK*-*lacZ* (del1 Cm^r) *ylxR* (Km^r)	This study
OAM854	*trpC2 amyE*::P*trmK*-*lacZ* (del2 Cm^r)	This study
OAM855	*trpC2 amyE*::P*trmK*-*lacZ* (del2 Cm^r) *ylxR* (Km^r)	This study
OAM856	*trpC2 amyE*::P*trmK*-*lacZ* (del3 Cm^r)	This study
OAM857	*trpC2 amyE*::P*trmK*-*lacZ* (del3 Cm^r) *ylxR* (Km^r)	This study
OAM858	*trpC2 amyE*::P*trmK*-*lacZ* (del4 Cm^r)	This study
OAM859	*trpC2 amyE*::P*trmK*-*lacZ* (del4 Cm^r) *ylxR* (Km^r)	This study
OAM860	*trpC2 amyE*::P*trmK*-*lacZ* (del5 Cm^r)	This study
OAM861	*trpC2 amyE*::P*trmK*-*lacZ* (del5 Cm^r) *ylxR* (Km^r)	This study
OAM862	*trpC2 amyE*::P*trmK*-*lacZ* (del6 Cm^r)	This study
OAM863	*trpC2 amyE*::P*trmK*-*lacZ* (del6 Cm^r) *ylxR* (Km^r)	This study
OAM864	*trpC2 amyE*::P*trmK*-*lacZ* (del6 Cm^r)	This study
OAM865	*trpC2 amyE*::P*trmK*-*lacZ* (del6 Cm^r) *ylxR* (Km^r)	This study
OAM866	*trpC2 amyE*::P*trmK*-*lacZ* (del7 Cm^r)	This study
OAM867	*trpC2 amyE*::P*trmK*-*lacZ* (del7 Cm^r) *ylxR* (Km^r)	This study
OAM868	*trpC2 amyE*::P*trmK*-*lacZ* (M1 Cm^r)	This study
OAM869	*trpC2 amyE*::P*trmK*-*lacZ* (M1 Cm^r) *ylxR* (Km^r)	This study
OAM870	*trpC2 amyE*::P*trmK*-*lacZ* (M2 Cm^r)	This study
OAM871	*trpC2 amyE*::P*trmK*-*lacZ* (M2 Cm^r) *ylxR* (Km^r)	This study
OAM872	*trpC2 amyE*::P*trmK*-*lacZ* (M3 Cm^r)	This study
OAM873	*trpC2 amyE*::P*trmK*-*lacZ* (M3 Cm^r) *ylxR* (Km^r)	This study
OAM753	*trpC2 thrC*::P*thiL*-*lacZ* (del1 Em^r)	This study
OAM754	*trpC2 thrC*::P*thiL*-*lacZ* (del1 Em^r) *ylxR* (Em^r *lacZ*::Tc^r)	This study
OAM755	*trpC2 thrC*::P*thiL*-*lacZ* (del1 Em^r) *yqfO* (Em^r *lacZ*::Tc^r)	This study
OAM756	*trpC2 thrC*::P*thiL*-*lacZ* (del2 Sp^r)	This study
OAM757	*trpC2 thrC*::P*thiL*-*lacZ* (del2 Sp^r) *ylxR* (Em^r *lacZ*::Tc^r)	This study
OAM758	*trpC2 thrC*::P*thiL*-*lacZ* (del2 Sp^r) *yqfO* (Em^r *lacZ*::Tc^r)	This study
OAM784	*trpC2 thrC*::P*thiL*-*lacZ* (del3 Em^r)	This study
OAM785	*trpC2 thrC*::P*thiL*-*lacZ* (del3 Em^r) *ylxR* (Em^r *lacZ*::Tc^r)	This study
OAM786	*trpC2 thrC*::P*thiL*-*lacZ* (del3 Em^r) *yqfO* (Em^r *lacZ*::Tc^r)	This study
OAM787	*trpC2 thrC*::P*thiL*-*lacZ* (del4 Sp^r)	This study
OAM788	*trpC2 thrC*::P*thiL*-*lacZ* (del4 Sp^r) *ylxR* (Em^r *lacZ*::Tc^r)	This study
OAM789	*trpC2 thrC*::P*thiL*-*lacZ* (del4 Sp^r) *yqfO* (Em^r *lacZ*::Tc^r)	This study
OAM790	*trpC2 thrC*::P*thiL*-*lacZ* (del5 Sp^r)	This study
OAM791	*trpC2 thrC*::P*thiL*-*lacZ* (del5 Sp^r) *ylxR* (Em^r *lacZ*::Tc^r)	This study
OAM792	*trpC2 thrC*::P*thiL*-*lacZ* (del5 Sp^r) *yqfO* (Em^r *lacZ*::Tc^r)	This study
OAM793	*trpC2 thrC*::P*thiL*-*lacZ* (del6 Sp^r)	This study
OAM794	*trpC2 thrC*::P*thiL*-*lacZ* (del6 Sp^r) *ylxR* (Em^r *lacZ*::Tc^r)	This study

OAM795	*trpC2 thrC*::P*thiL-lacZ* (del6 Sp^{r}) *yqfO* (Em^{r} *lacZ*::Tc^{r})	This study
OAM796	*trpC2 thrC*::P*thiL-lacZ* (del7 Sp^{r})	This study
OAM797	*trpC2 thrC*::P*thiL-lacZ* (del7 Sp^{r}) *ylxR* (Em^{r} *lacZ*::Tc^{r})	This study
OAM798	*trpC2 thrC*::P*thiL-lacZ* (del7 Sp^{r}) *yqfO* (Em^{r} *lacZ*::Tc^{r})	This study
OAM799	*trpC2 thrC*::P*thiL-lacZ* (del8 Sp^{r})	This study
OAM800	*trpC2 thrC*::P*thiL-lacZ* (del8 Sp^{r}) *ylxR* (Em^{r} *lacZ*::Tc^{r})	This study
OAM801	*trpC2 thrC*::P*thiL-lacZ* (del8 Sp^{r}) *yqfO* (Em^{r} *lacZ*::Tc^{r})	This study
OAM802	*trpC2 thrC*::P*thiL-lacZ* (M1 Sp^{r})	This study
OAM803	*trpC2 thrC*::P*thiL-lacZ* (M1 Sp^{r}) *ylxR* (Em^{r} *lacZ*::Tc^{r})	This study
OAM804	*trpC2 thrC*::P*thiL-lacZ* (M1 Sp^{r}) *yqfO* (Em^{r} *lacZ*::Tc^{r})	This study
Plasmid	Description	Reference or source
pDH88	Amp^{r} Pspac Cm^{r}	Henner, 1990
pDH88-ykqC	Amp^{r} Pspac-*ykqC* (N-terminal region of *rnjA*) Cm^{r}	This study
pMUTIN	Amp^{r} Em^{r} *lacZ* Pspac	Vagner *et al*., 1998
pMUTIN-pdhD	Amp^{r} Em^{r} *pdhD*-*lacZ* Pspac	This study
pSG1154	Amp^{r} Sp^{r} *amyE*::Pxyl-*gfp*	Lewis and Marston, 1999
pSG1154-pdhA	Amp^{r} Sp^{r} *amyE*::Pxyl-*pdhA*-*gfp*	This study
ECE74	Amp^{r} Cm^{r}::Sp^{r}	BGSC
pIS-trmK-del1	Amp^{r} *amyE*::P*trmK-lacZ* (-220 to -1[2]) Cm^{r}	This study
pIS-trmK-del2	Amp^{r} *amyE*::P*trmK-lacZ* (-174 to -1) Cm^{r}	This study
pIS-trmK-del3	Amp^{r} *amyE*::P*trmK-lacZ* (-147 to -1) Cm^{r}	This study
pIS-trmK-del4	Amp^{r} *amyE*::P*trmK-lacZ* (-94 to -1) Cm^{r}	This study
pIS-trmK-del5	Amp^{r} *amyE*::P*trmK-lacZ* (-220 to -95) Cm^{r}	This study
pIS-trmK-del6	Amp^{r} *amyE*::P*trmK-lacZ* (-295 to -95) Cm^{r}	This study
pIS-trmK-del7	Amp^{r} *amyE*::P*trmK-lacZ* (-297 to -41) Cm^{r}	This study
pIS-trmK-M1	Amp^{r} *amyE*::P*trmK-lacZ* (-297 to -1) carrying nt change, Cm^{r}	This study
pIS-trmK-M2	Amp^{r} *amyE*::P*trmK-lacZ* (-297 to -1) carrying nt change, Cm^{r}	This study
pIS-trmK-M3	Amp^{r} *amyE*::P*trmK-lacZ* (-297 to -1) carrying nt change, Cm^{r}	This study
pDG1663	Amp^{r} Sp^{r} *thrC*::*lacZ* Em^{r}	Guerout-Fleury *et al*., 1996
pDG1663-thiL-del1	Amp^{r} Sp^{r} *thrC*::P*thiL-lacZ* (-297 to -1[2]) Em^{r}	This study
pDG1663-thiL-del3	Amp^{r} Sp^{r} *thrC*::P*thiL-lacZ* (-138 to -1) Em^{r}	This study
pDG1729-thiL-del2	Amp^{r} Em^{r} *thrC*::P*thiL-lacZ* (-210 to -1) Sp^{r}	This study
pDG1729-thiL-del4	Amp^{r} Em^{r} *thrC*::P*thiL-lacZ* (-500 to -278) Sp^{r}	This study
pDG1729-thiL-del5	Amp^{r} Em^{r} *thrC*::P*thiL-lacZ* (-500 to -170) Sp^{r}	This study
pDG1729-thiL-del6	Amp^{r} Em^{r} *thrC*::P*thiL-lacZ* (-500 to -137) Sp^{r}	This study
pDG1729-thiL-del7	Amp^{r} Em^{r} *thrC*::P*thiL-lacZ* (-500 to -68) Sp^{r}	This study
pDG1729-thiL-del8	Amp^{r} Em^{r} *thrC*::P*thiL-lacZ* (-500 to -39) Sp^{r}	This study
pDG1729-thiL-M1	Amp^{r} Em^{r} *thrC*::P*thiL-lacZ* (-500 to -1) carrying nt change, Sp^{r}	This study

[1] Numbers indicate the nucleotide positions ralative to the transcription start point for *sigX* .

[2] Numbers indicate the nucleotide positions ralative to the translation start point for *thiL and trmK*.

Table S2. Oligonucleotides used for this study.

Name	Sequence	Product/use
pX-yqfO-Spe	5'-AAACTAGTTAAAGGAGGTAATCGATCATGGC-3'	pX-yqfO
pX-yqfO-BglI	5'-ATGAGATCTTTATAGAAATGTAAATGGATTTGTATCTG-3'	pX-yqfO
pX-gcp-Spe	5'-AAACTAGTAATTATGTGGGTGACGATAAATG-3'	pX-tsaD
pX-gcp-Bam	5'-ATGGGATCCTTATCTCGTGAGACTTTGATAAG-3'	pX-tsaD
pdhA-F-E	5'-CGCGAATTCTGTAGCTGCCGGTATTGTCGG-3	pMUTIN-His-pdhA
pdhA-R-Xh	5'-GCGCTCGAGCTTCGACTCCTTCTGTGTATAAA-3'	pMUTIN-His-pdhA
pdhB-F-E	5'-CGCGAATTCCGTCAGGAAGTTCCTGAAGA-3'	pMUTIN-His-pdhB
pdhB-R-Xh	5'-GCGCTCGAGAAATTCAAGCACTTTTCTTGCTGTTTC-3'	pMUTIN-His-pdhB
pdhD-F-E	5'-CGCGAATTCCCGCTTGCTCATAAAGCATC-3'	pMUTIN-His-pdhD
pdhD-R-Xh	5'-GCGCTCGAGTTTTACGATGTGAATCGGACTTCC-3'	pMUTIN-His-pdhD
pMut-pdhD-E	5'-ATTGAATTCGGTGATATCATCGAAGGA-3'	pMUTIN-pdhD
pMut-pdhD-B	5'-ATCGGATCCGATATGAAAATTATTTTACGATGTG-3'	pMUTIN-pdhD
pSG1154-pdhA-Xh	5'-ATTCTCGAGCGATGAAAACGATTGACGGTTC-3'	pSG1154-pdhA
pSG1154-pdhA-Ec	5'-ATTGAATTCCTTCGACTCCTTCTGTGTATAAA-3'	pSG1154-pdhA
YkqC-3	5’-TGGAAGCTTTTAAAGTATTGGAGTTATGA-3’	pDH88-ykqC
YkqC-4	5’-TGGTCTAGATTATTCCGAAGTAAGCCGAT-3’	pDH88-ykqC
acsA-FF	5'-TTCCGACAGCGCATCCCCGA--3'	acsA (Tc^r)
acsA(Tc)-FR	5'-GCTGTTCATATCGACCCTCTGCCTCGGCCCAATCAA-3'	acsA (Tc^r)
acsA(Tc)-RF	5'-TTTTTTATAACAGGAATTCGGGTCTTGCAGCCCATGCGGC-3'	acsA (Tc^r)
acsA-RR	5'-AATAGGCAGCAGGTGTCCGA-3'	acsA (Tc^r)
pta-FF	5'-TTTGGCCGACGATGCTGTAA-3'	pta (Tc^r)
pta (Tc)-FR	5'-GCTGTTCATATCGACCCGCTCGTCTAAGCCTTCAGG-3'	pta (Tc^r)
pta (Tc)-RF	5'-TTTTTTATAACAGGAATTCGGATGTAACGCTGAAGATGT-3'	pta (Tc^r)
pta-RR	5'-GCAGCTGGGCCAATAGCGG-3'	pta (Tc^r)
ydiC-FF	5'-GACGGGTGACTCGTGTGAAGC-3'	tsaB (Tc^r)
ydiC(Tc)-FR	5'-GCTGTTCATATCGACCCGAAGCAGCGCAATGCCCAA-3'	tsaB (Tc^r)
ydiC(Tc)-RF	5'-TTTTTTATAACAGGAATTCCTTCTCTTCACAAGCAGATGA--3'	tsaB (Tc^r)
ydiC-RR	5'-CTTCCCCGTTATCAGTATAATAG-3'	tsaB (Tc^r)
TC-F	5'-GGTCGATATGAACAGCTTATTTAC-3'	acsA (Tc^r), ylxQ (Tc^r), tsaB (Tc^r), pta (Tc^r)
TC-R	5'-GAATTCCTGTTATAAAAAAAGGATCAA-3'	acsA (Tc^r), ylxQ (Tc^r), tsaB (Tc^r), pta (Tc^r)
ackA-FF	5'-CATGCCAAAAGCATCCTTGTG-3'	ackA (Sp^r)
ackA (Sp)-FR	5'-CCAGTCACGTTACGTAAGACGAGCTTCCTGCGTTA-3'	ackA (Sp^r)
ackA (Sp)-RF	5'-CTAATTGGTAATCAGACCCGACTGATGAAGAAGTCATG-3'	ackA (Sp^r)
ackA-RR	5'-ACAGCGGTATGCTGAATGAC-3'	ackA (Sp^r)
Spc-F	5'-ACGTAACGTGACTGGCAAGA-3'	ackA (Sp^r)
Spc-R	5'-TCTGATTACCAATTAGAATGAAT-3'	ackA (Sp^r)
yqfO-Eco	5'-AATGAATTCTGGATGATATGGCAGAGTGG-3'	pIS-trmK, -del6/7, M2/3
pIS-trmK-B	5-ATCGGATCCACCGCTCCATTCGGTATGT-3	pIS-trmK, -del1/2/3/4, M1/2/3
pIS-trmK-E-del1	5-AATGAATTCGAGATGTTCTCGACAGCCT-3	pIS-trmK-del1, -del5
pIS-trmK-E-del1.1	5-AATGAATTCCGCATCAAAAGAAATAATTTCAAAATC-3	pIS-trmK-del2
pIS-trmK-E-del2	5-AATGAATTCATTTGCTTCAGCTTGGATTCC-3	pIS-trmK-del3
pIS-trmK-E-del3	5-AATGAATTCAAATGATTGAGAAAAGTTGTGATGA-3	pIS-trmK-del4
pIS-trmK-B-del1	5-AATGAATTCTTTCCTTTACTATCATATAAAAAATC-3	pIS-trmK-del5, -del6
pIS-trmK-B-del1.1	5-ATCGGATCCGTTTAGATAATTTTAATTCGTTCAC-3	pIS-trmK-del7
yqfO-M6	5-AATGAATTCTGGATGATATGGCAGAGTGGGTGTACCAAATTAA-3	pIS-trmK-M1
pIS-trmK-M1a	5-GCAGACCCGCATACCCAGAAATAATTTC-3	pIS-trmK-M2
pIS-trmK-M1b	5-GAAATTATTTCTGGGTATGCGGGTCTGC-3	pIS-trmK-M2
pIS-trmK-M2a	5-AAAGAAATAATTTACCCATCATTTGCTTCAG-3	pIS-trmK-M3
pIS-trmK-M2b	5-CTGAAGCAAATGATGGGTAAATTATTTCTTT-3	pIS-trmK-M3, EMSA (-295/-135, M3, M1/3)
pDG1729-gcp-E	5'-ATTGAATTCACACCCGTTCCCATACCGAACA-3'	pDG1729-thiL-Wt, -del4/5/6/7/8, -M1
pDG1729-gcp-B	5'-ATCGGATCCGAACCAAAACCTCCTTTCAACC-3'	pDG1729-thiL-Wt, -del1/2/3, -M1
pDG1729-gcp-E2	5'-ATTGAATTCGTGCCCTAAGGGTGCAACCA-3'	pDG1663-thiL-del1
pDG1729-gcp-E2.53	5'-ATTGAATTCTCGCGGGTTCGAGTCCCGTC-3'	pDG1739-thiL-del2
pDG1729-gcp-E3	5'-ATTGAATTCTTTTACTGTATCTTCTGCTTGGTG-3'	pDG1663-thiL-del3
gcp-R2	5'-ATCGGATCCTGGTTGCACCCTTAGGGCAC-3'	pDG1729-thiL-del4
pDG1729-gcp-B2	5'-ATTGGATCCGTTTACATTAATGGCGGTCCGG-3'	pDG1729-thiL-del5
pDG1729-gcp-B3	5'-ATTGGATCCAAAACACAAAAAAAACGAAAACATGATCG-3'	pDG1729-thiL-del6
pDG1729-gcp-B4	5'-ATTGGATCCGGAATATCCAAGATGTTCCCGAT-3'	pDG1729-thiL-del7
pDG1729-gcp-B5	5'-ATTGGATCCGTGCTCTTCCTAGTTTATCGAA-3'	pDG1729-thiL-del8
pDG1729-thiL-M1	5'-CTTGGATATTCCAtaccAATTCGATAAACTAGG-3'	pDG1729-thiL-M1
pDG1729-thiL-M2	5'-CCTAGTTTATCGAATTggtaTGGAATATCCAAG-3'	pDG1729-thiL-M1

yqfO-EMSA1	5-CTGAAGCAAATGATTTTGAAATTATTTC-3	EMSA (-295/-135, Wt, M1)
yqfO-F-bio	5'-biotin-CTGGATGATATGGCAGAGTGG-3-3'	EMSA (-295/-135, Wt, M1, M3, M1/3)
gcp-biotin-R	5'-biotin-GAACCAAAACCTCCTTTCAACC-3'	EMSA (-76/-1, Wt)(-76/-1, M1)
thiL-F4	5-GGATATTCCAGCAAAATTCGATAAA-3	EMSA (-76/-1, Wt)
thiL-F4M	5-GGATATTCCAtaccAATTCGATAAACTAGG-3	EMSA (-76/-1, M1)

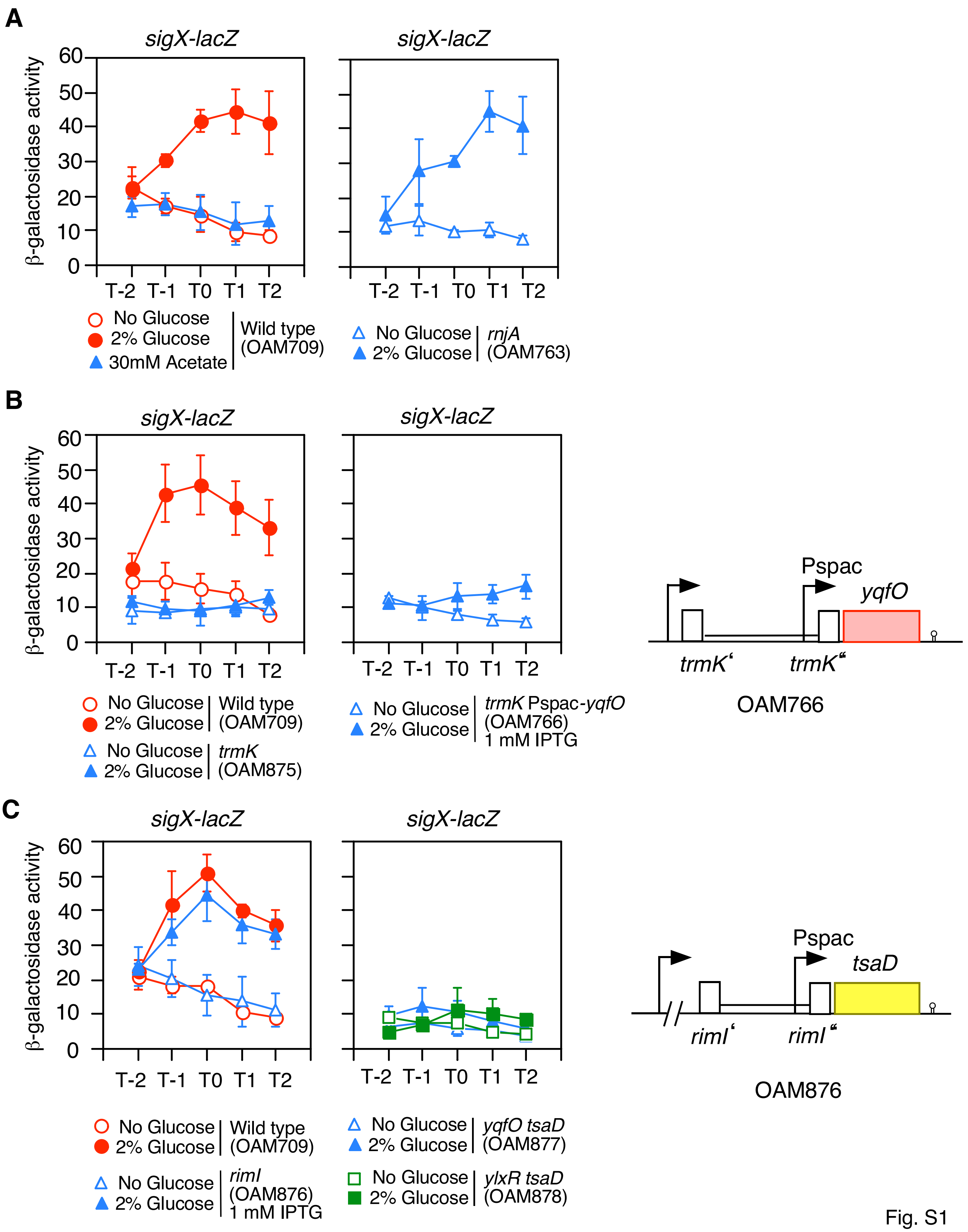

A
B
C
sigX-lacZ
β-galactosidase activity
0
10
20
30
40
50
60
T-2
T-1
T0
T1
T2
No Glucose
2% Glucose
30mM Acetate
Wild type
(OAM709)
rnjA
(OAM763)
trmK
(OAM875)
trmK Pspac-yqfO
(OAM766)
1 mM IPTG
Pspac
yqfO
trmK'
trmK"
OAM766
rimI
(OAM876)
yqfO tsaD
(OAM877)
ylxR tsaD
(OAM878)
tsaD
rimI'
rimI"
OAM876

Fig. S1

A

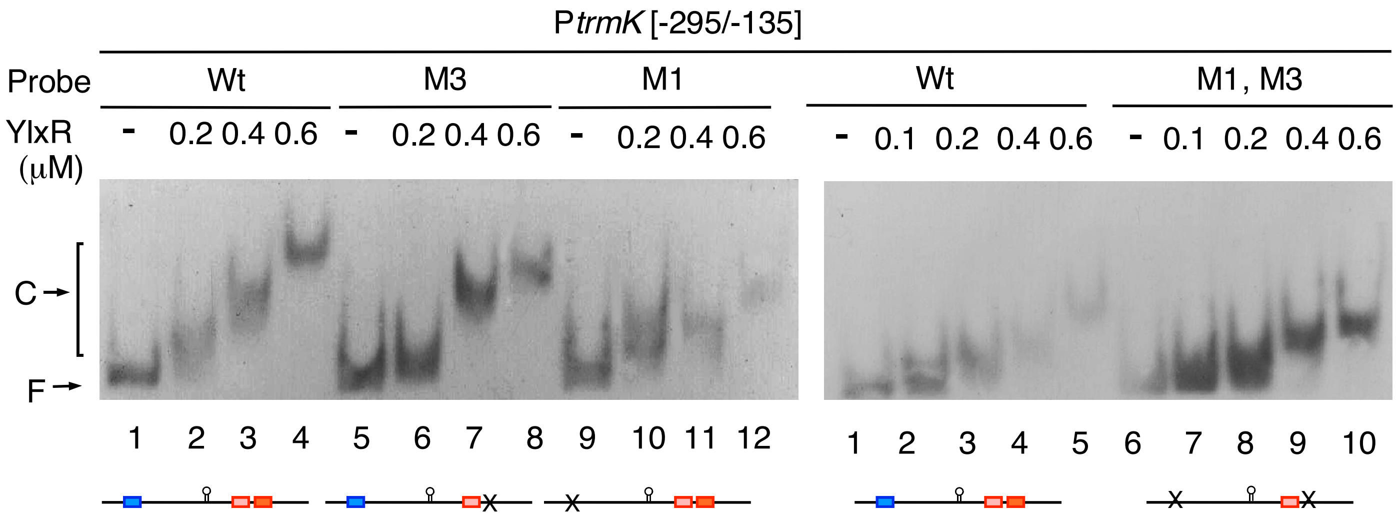
PtrmK [-295/-135]
Probe
Wt
M3
M1
Wt
M1, M3
YlxR (μM)
- 0.2 0.4 0.6
- 0.2 0.4 0.6
- 0.2 0.4 0.6
- 0.1 0.2 0.4 0.6
- 0.1 0.2 0.4 0.6
C
F
1 2 3 4 5 6 7 8 9 10 11 12
1 2 3 4 5 6 7 8 9 10

B

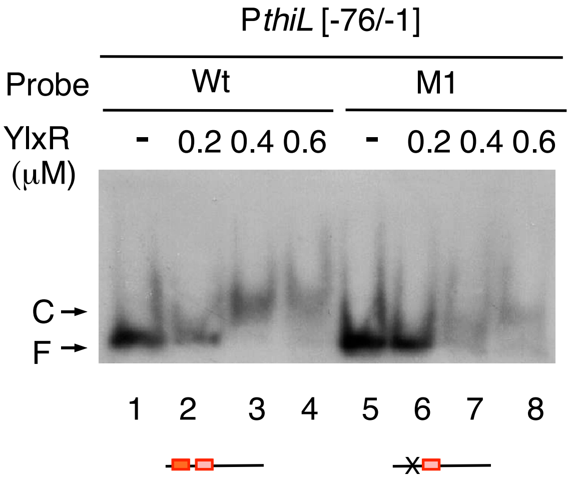
PthiL [-76/-1]
Probe
Wt
M1
YlxR (μM)
- 0.2 0.4 0.6
- 0.2 0.4 0.6
C
F
1 2 3 4 5 6 7 8

Fig. S2

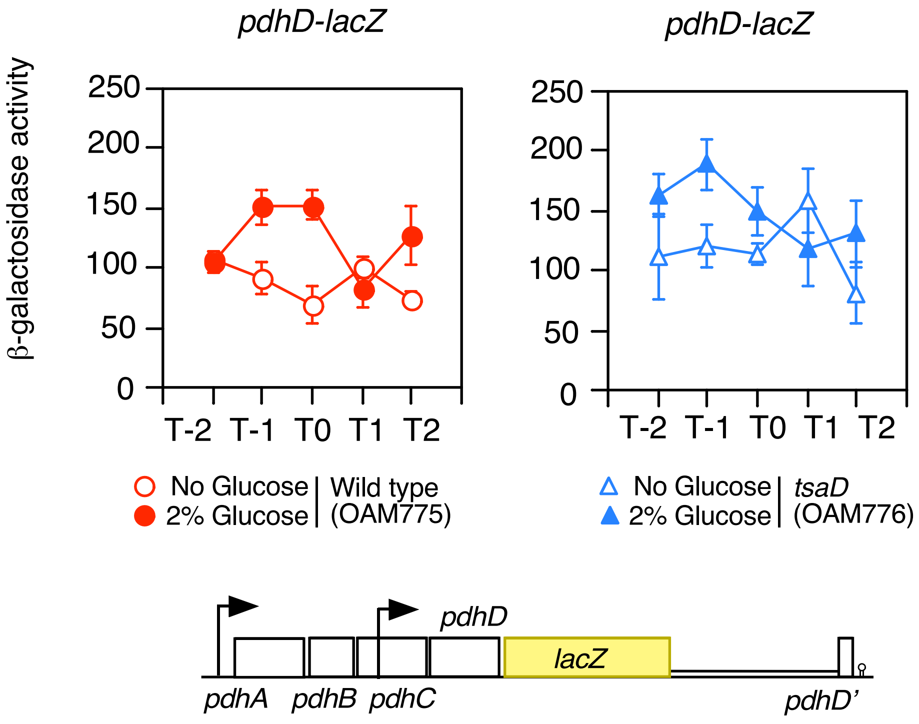

pdhD-lacZ
pdhD-lacZ
β-galactosidase activity
250
200
150
100
50
0
T-2 T-1 T0 T1 T2
No Glucose
2% Glucose
Wild type
(OAM775)
No Glucose
2% Glucose
tsaD
(OAM776)
pdhD
lacZ
pdhA
pdhB
pdhC
pdhD'

Fig. S3

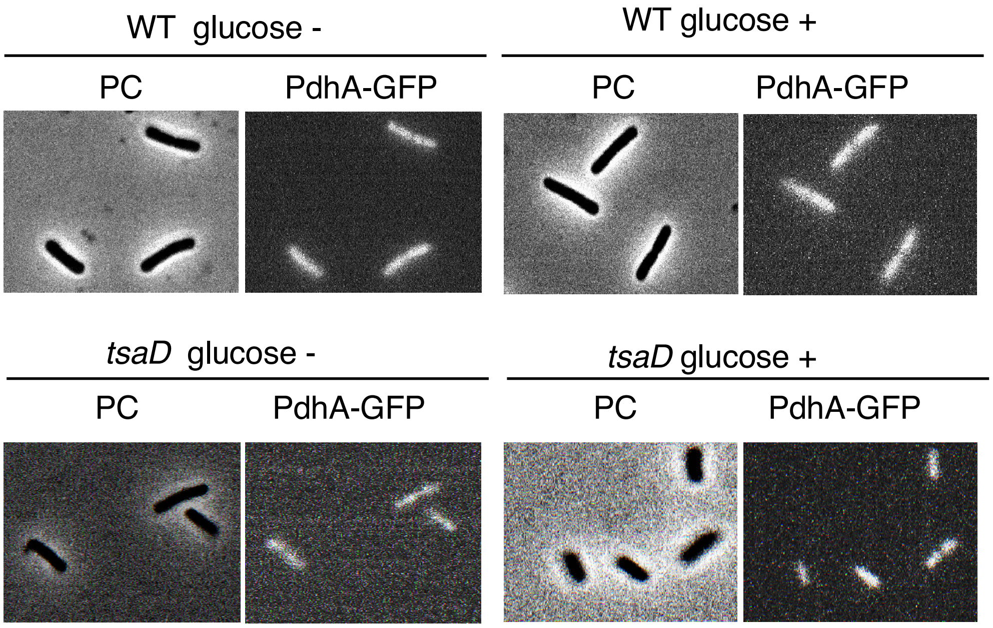
WT glucose -
PC
PdhA-GFP
WT glucose +
PC
PdhA-GFP
tsaD glucose -
PC
PdhA-GFP
tsaD glucose +
PC
PdhA-GFP

Fig. S4

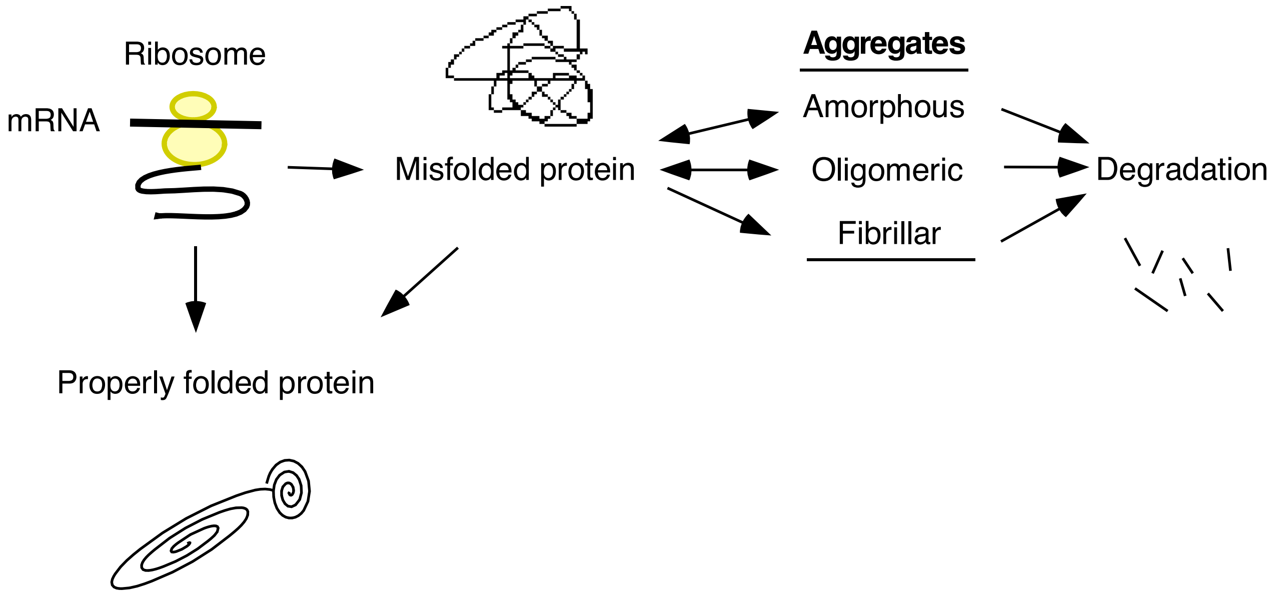

Ribosome
mRNA
Misfolded protein
Aggregates
Amorphous
Oligomeric
Fibrillar
Degradation
Properly folded protein

Fig. S5
